# A Passive Perspiration Inspired Wearable Platform for Continuous Glucose Monitoring

**DOI:** 10.1002/advs.202405518

**Published:** 2024-09-12

**Authors:** Tamoghna Saha, Muhammad Inam Khan, Samar Singh Sandhu, Lu Yin, Sara Earney, Chenyang Zhang, Omeed Djassemi, Zongnan Wang, Jintong Han, Abdulhameed Abdal, Samarth Srivatsa, Shichao Ding, Joseph Wang

**Affiliations:** ^1^ Aiiso Yufeng Li Family Department of Chemical and Nanoengineering University of California San Diego La Jolla CA 92093 USA; ^2^ Department of Bioengineering University of California San Diego La Jolla CA 92093 USA; ^3^ Department of Mechanical Engineering University of California San Diego La Jolla CA 92093 USA

**Keywords:** biomarker, continuous glucose monitoring, electrodes, sweat, wearable sensor

## Abstract

The demand for glucose monitoring devices has witnessed continuous growth from the rising diabetic population. The traditional approach of blood glucose (BG) sensor strip testing generates only intermittent glucose readings. Interstitial fluid‐based devices measure glucose dynamically, but their sensing approaches remain either minimally invasive or prone to skin irritation. Here, a sweat glucose monitoring system is presented, which completely operates under rest with no sweat stimulation and can generate real‐time BG dynamics. Osmotically driven hydrogels, capillary action with paper microfluidics, and self‐powered enzymatic biochemical sensor are used for simultaneous sweat extraction, transport, and glucose monitoring, respectively. The osmotic forces facilitate greater flux inflow and minimize sweat rate fluctuations compared to natural perspiration‐based sampling. The epidermal platform is tested on fingertip and forearm under varying physiological conditions. Personalized calibration models are developed and validated to obtain real‐time BG information from sweat. The estimated BG concentration showed a good correlation with measured BG concentration, with all values lying in the A+B region of consensus error grid (MARD = 10.56% (fingertip) and 13.17% (forearm)). Overall, the successful execution of such osmotically driven continuous BG monitoring system from passive sweat can be a useful addition to the next‐generation continuous sweat glucose monitors.

## Graphical abstract

A passive perspiration enabled wearable platform for continuous blood glucose tracking. The platform utilizes osmosis for non‐invasive epidermal sweat extraction and an electrochemical self‐powered sensor to measure glucose. This is the first demonstration of a complete zero electric powered (extraction and sensing) sweat glucose patch, with an overall mean absolute relative difference of ~ 11% vs. blood glucose meter.

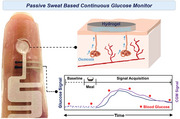

## 1 Introduction

Diabetes is a major global health concern since nearly 450 million people (≈5% of world population) are reported to be currently impacted by it, with a resulting cumulative health expenditure of ≈$950–1050 billion till date^[^ ^1^, ^2^, ^3^ ^]^ Type‐1 (T1D) and type‐2 (T2D) diabetes are the common forms of the disease, where T1D is associated with the deficiency of insulin producing β cells of the pancreas, while T2D occurs due to abnormal insulin secretion and insulin resistance (incapability of insulin to efficiently metabolize carbohydrate).^[^ ^1^ ^]^ The biomarkers targeted for diabetes diagnosis are glucose and glycated hemoglobin (HbA1c), and blood is used as the primary biofluid to quantify them. However, sampling blood frequently for long term is not feasible (due to fingerpicking). Alternatively, over the past decade researchers have actively explored and made significant progress toward monitoring glucose levels in other biofluid (such as interstitial fluid (ISF), saliva, tears, and sweat).^[^ ^2^ ^]^ Amongst these, ISF has proven to be the best candidate for glucose estimation due to its close proximity with the blood capillaries and good correlation with blood glucose (BG) concentration.^[^ ^4^, ^5^ ^]^ The correlation reports between glucose levels in blood with saliva and tears have mixed reviews.^[^ ^1^ ^]^ Although ISF is a suitable candidate, accessing it can be achieved only with devices such as microneedles^[^ ^6^ ^]^ or with sampling techniques such as reverse iontophoresis (RI).^[^ ^7^ ^]^ Commercially available continuous glucose monitors (CGMs) also rely on needle‐based on‐tip sensing to estimate dynamic glucose levels (from sub dermal zone). Such minimally invasive devices are fully integrated systems which can continuously monitor glucose for up to 14 days.^[^ ^8^, ^9^ ^]^ CGMs are typically expensive (≈$150‐500/month without health insurance) and rely on advanced fabrication strategies. RI‐based ISF glucose sensing involves driving current through the skin surface which can cause skin irritation. Thus, there remains a necessity to explore continuous glucose measurements in alternative biofluids which may be easier to access on the skin surface and could support continuous monitoring.^[^ ^1^ ^]^ Monitoring from sweat can potentially address these challenges due to a) the wide distribution of sweat glands throughout the body (≈2–4 million in total) ^[^ ^10^ ^]^ b) it being a rich repository of several biomarkers^[^ ^10^, ^11^ ^]^ and c) easy generation on the skin surface through elevated core body temperature (exercise, sauna)^[^ ^10^, ^12^ ^]^ However, since glucose exists in sub millimolar range in sweat^[^ ^11^ ^]^, monitoring it under high perspiration rates is not feasible (due to dilution and correlation error with blood), while monitoring at low perspiration rates is challenging from the analytical and engineering points of view.

Sweat based electrochemical glucose biosensors have been actively investigated since the mid 2010′s.^[^ ^11^, ^13^ ^]^ The field has progressed significantly over years toward continuous wearable systems.^[^ ^14^, ^15^, ^16^, ^17^, ^18^, ^19^, ^20^, ^21^, ^22^, ^23^, ^24^, ^25^, ^26^, ^27^, ^28^ ^]^ Such systems function on either exercise^[^ ^13^ ^]^ or with chemically‐induced sweat^[^ ^25^ ^]^ (via pilocarpine or carbachol transdermal delivery) on skin. However, the correlation between blood and estimated sweat glucose concentration from these techniques have mixed reviews, with some claiming a good correlation^[^ ^14^, ^18^, ^22^, ^25^ ^]^ while the others do not.^[^ ^13^, ^28^ ^]^ Such reports are attributed to the partitioning mechanism of glucose from blood to sweat,^[^ ^29^ ^]^ interpersonal sweat rate variations, and improper sweat sampling techniques.^[^ ^28^ ^]^ The estimated sweat glucose concentration in some prototypes can be corrected by accounting for sweat pH and temperature fluctuations,^[^ ^14^, ^28^ ^]^ but execution of these strategies require considerable sweat volume (> 100 µL). Handling excessive sweat volume raises concerns regarding contamination, dilution, and losses from evaporation. To address these issues, researchers have recently introduced hydrogels as a potential tool to deal with low sweat volumes.^[^ ^30^, ^31^, ^32^ ^]^ This concept remains executed for touch‐based sensing from fingertip sweat at rest,^[^ ^31^, ^33^, ^34^ ^]^ where the hydrogel drives the analyte transport from the skin to the sensor via diffusion and capillarity. Previous touch‐based sweat assays have targeted measuring glucose,^[^ ^34^ ^]^ lactate,^[^ ^31^ ^]^ β‐hydroxybutyrate,^[^ ^32^ ^]^ and levodopa.^[^ ^35^ ^]^ However, this technique generates only periodic readings and cannot track dynamic glucose fluctuations. Hence, there remains an urgent need for a sweat‐based continuous glucose monitoring (SCGM) platform, which can operate at rest and address all the above limitations.

**Figure 1:**
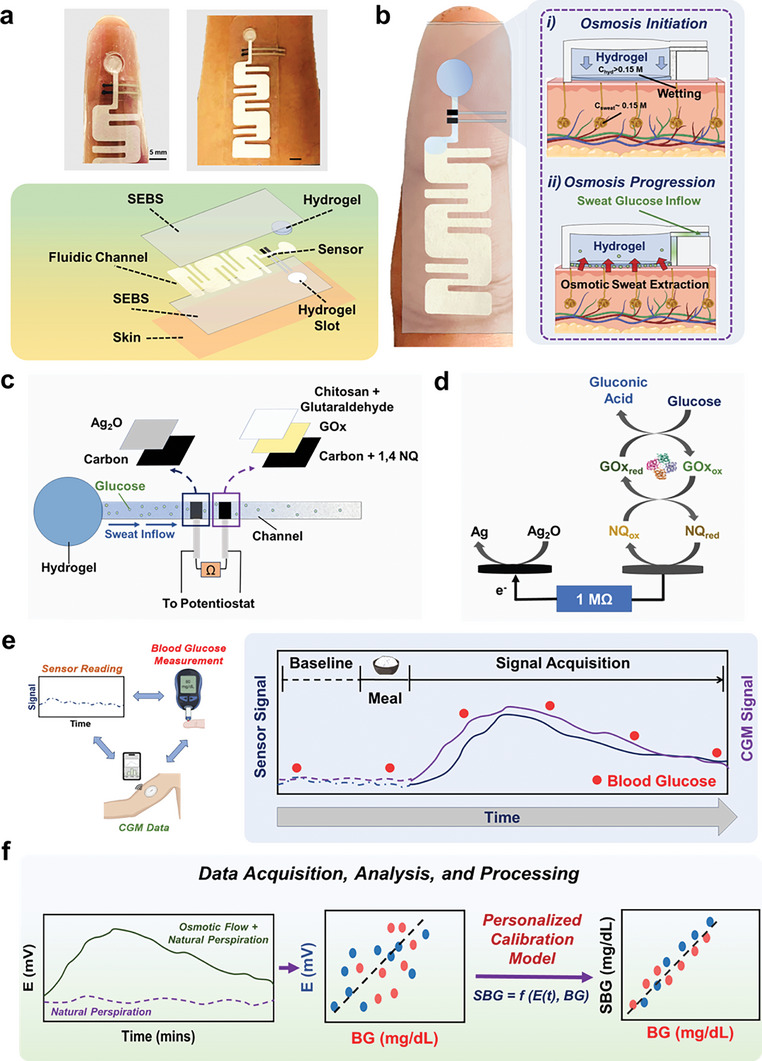
Osmotic sweat extraction based wearable glucose monitoring system. A) Optical image of the wearable platform on the fingertip and forearm along with the exploded view schematic. B) Schematic highlighting the interfacing of the platform with the skin and the operating principle of the sweat extraction via osmosis. C) The composition of the potentiometric glucose sensor and how it is interfaced onto the paper channel to measure glucose from the inflowing sweat. D) Sensing mechanism of the self‐powered potentiometric glucose sensor. E) The workflow of sweat glucose detection on body, which involves measuring the sensor baseline, consuming meal, and observing the sweat, blood, and CGM (only diabetic subjects) glucose trends over time. F) Schematic highlighting the synergistic effect added by osmotic forces toward enhanced sweat glucose detection. The acquired raw sensor data is further processed using personalized calibration models in each subject to obtain accurate sweat‐based blood glucose concentration values.

In this study, we introduce a wearable SCGM platform that synergistically utilizes osmosis to enhance sweat extraction (in addition to natural perspiration), capillary action for sweat transport and management, and a self‐powered glucose biosensor for continuous sensing (**Figure** **1**a). The osmotic effect is deployed on skin with a high solute‐containing hydrogel, while the sweat transport is achieved using a paper microfluidic channel. Such use of osmosis is shown to facilitate greater sweat collection compared to natural perspiration alone, which can be executed at different body locations by tuning the hydrogel composition and osmolyte strength. All components are encased using a stretchable styrene‐ethylene‐butylene‐styrene (SEBS) thermoplastic elastomeric substrate, which prevents the drying of the hydrogel and paper, while allowing conformal adherence of the platform onto the fingertip and forearm, both known for their high sweat gland density.^[^ ^36^ ^]^ The paper channel stays sandwiched between the electrode and top SEBS cover, and comprises of two parts: a circular section to host the hydrogel and directly interface the skin, and a rectangular section with a large surface area (7.2 cm^2^) to facilitate the prolonged sweat flow via capillary action and prevent the intermixing with old sweat. The glucose biosensor interfaces the thin rectangular section of the paper channel near the inlet (hydrogel site) to instantaneously detect any inflowing sweat glucose and avoid flux losses arising from chromatographic effects of paper. In our case, we use ethylene glycol (EG) as the osmolyte to equilibrate the hydrogels. EG not only helps in raising the osmotic pressure of the hydrogel with respect to sweat but also prevents the hydrogel from drying due to its non‐volatile nature without affecting the sensor performance. As the platform interfaces the skin, the hydrogel slightly wets the circular section of the paper channel with EG (Figure 1b). This first removes the dead volume space in the channel, and second builds up the osmotic pressure difference (vs sweat) to drive the sweat flow (including naturally perspired sweat) from the skin toward the sensor. Moreover, having an inherent hydrophilic material, like paper‐ a) facilitates easy sweat collection compared to conventional microfluidic channel‐based platforms, where flow would only occur if the hydrostatic pressure of the sweat overcomes the hydrodynamic resistance in the channel (Note S1, Supporting Information), b) prevents intermixing of old and new sweat due to capillary wicking in the forward direction.^[^ ^37^, ^38^ ^]^ Furthermore, osmosis – being a colligative property – remains independent of pH and temperature fluctuations, and would sustain the sweat collection as long as the chemical potential difference of solute and solvent is maintained between the gel and sweat. Therefore, unlike iontophoresis (which operates with ≈0.2‐1 mA cm^−2^), this approach offers an exertion‐free and zero‐power consuming route for epidermal sweat extraction. The sampled sweat glucose gets detected by a self‐powered, second‐generation (mediator‐based and oxygen‐independent) enzymatic glucose biosensor (Figure 1c), which makes SCGM a fully low‐powered platform (for both sweat extraction and sensing), unlike the previously reported devices.^[^ ^30^, ^39^ ^]^ The sensor comprises a two‐electrode system and relies on its intrinsic potential difference to operate, unlike controlled‐potential amperometric measurements. The anode uses a porous carbon ink along with 1,4 napthaquinone (NQ) mediated oxidation of glucose by the glucose oxidase enzyme (GOx), with open circuit potential (OCP) ≈−0.15 V versus Ag/AgCl (Figure 1d). The cathode is developed using Ag_2_O (OCP ≈+0.27 V vs Ag/AgCl) which reduces to Ag. An external load of 1MΩ drives the current flow between the two electrodes, which is dominated by the concentration‐limited electrocatalytic reaction on the GOx electrode. The external load was fixed based on the obtained signal resolution from linear sweep voltammetry with multiple glucose additions in‐vitro (Figure S1, Supporting Information). The on‐body SCGM operation was coupled to parallel BG concentration measurements (via fingerpick) in healthy subjects and with commercial CGM monitor readouts in diabetic subjects (Figure 1e). All subjects underwent overnight fasting and wore the SCGM the next day. BG concentration, the sensor, and CGM readouts get initially noted at the fasting state. Next, all subjects consumed the same meal (to fix carbohydrate bolus) and monitored these three parameters for the next 2—5 h. The resulting potentiometric SCGM signals are subsequently processed using a personalized calibration model to obtain an equivalent sweat‐based blood glucose (SBG) concentration at each time stamp for every subject (Figure 1f). The parameters (slope and intercept) used in these individualized models are evaluated based on the net change in potential, and blood glucose concentration during the test. The consistency of the parameters is evaluated through multiple trials over extended periods. Careful evaluation of these parameters contributes toward: a) accurate estimation of SBG in every individual and b) reducing the frequency of repeated blood‐based calibrations. The correlation between the SBG concentration, measured BG concentration, and CGM data is then assessed statistically to analyze the SCGM performance and accuracy on both healthy and diabetic subjects. Such passive perspiration‐based SCGM operation with personalized corrections allows real‐time tracking of BG dynamics (via SBG estimation) along with improved correlations versus the measured fingerpick BG concentration.

## 2 Results and Discussion

### 2.1 Optimizing the Hydrogel Composition Toward Continuous Sweat Extraction

**Figure 2:**
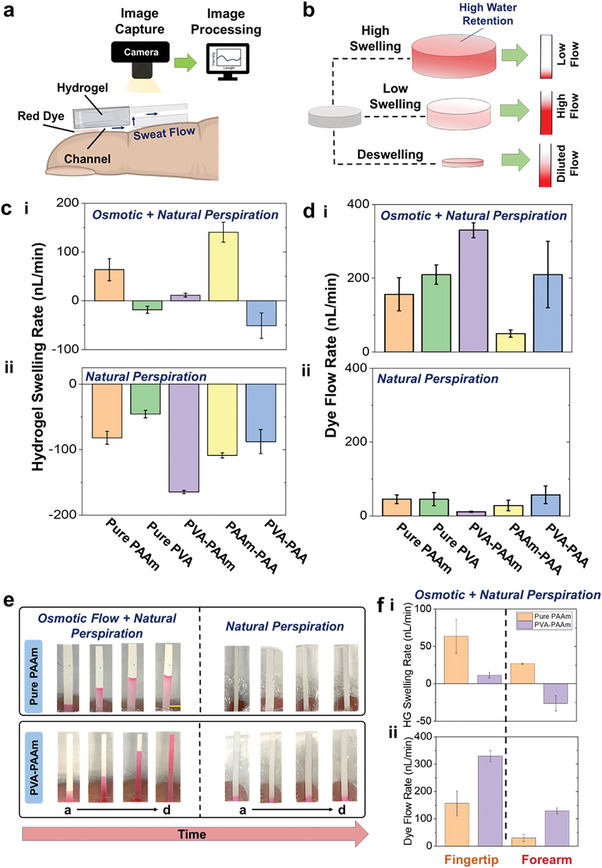
On‐skin investigation of the hydrogel performance toward continuous sweat extraction. A) Schematic depicting how the performance of a hydrogel toward sweat withdrawal is examined on the fingertip. Using various hydrogels in the patch, the transport of red dye (model biomarker) is tracked on the paper fluidic channel using image analysis. B) Schematic depicting how the hydrogel swelling is related to the intensity of the sampled dye on the paper channel. C) Plot showing the swelling rate of five different hydrogels on the fingertip under **i** combination of osmotic and natural perspiration, and **ii** natural perspiration. **D)** Plot with the measured dye flow rate as obtained using five different hydrogels on the fingertip under **i** combination of osmotic and natural perspiration, and using **ii** natural perspiration alone. **E)** Optical images of the dye‐stained paper channels for the two best performing hydrogels on fingertip for 2 h. Scale 2 mm. **f** Plot showing the **i** hydrogel swelling rate and the **ii** dye flow rate for pure PAAm (orange) and PVA‐PAAm (purple) hydrogels on the forearm. The bar plots represent mean ± standard deviation (S.D.), and all error bars denote S.D. from n = 4 trials.

The hydrogel composition was first investigated in vitro before transitioning to on‐body trials. For this, the circular section of the paper channel in the platform was initially treated with a red dye (model biomarker) and tested with different hydrogel variants on the fingertip (like Figure 1a). The hydrogel swelling rate and the dye flow rate on paper were evaluated (via image analysis) to analyze the hydrogel performance (**Figure** **2**a). Our goal was to compare the sweat withdrawing capacity of different hydrogel variants, with (EG treated) and without (natural perspiration, 0.1 M PBS treated) the effect of osmosis. Fingertip was initially chosen as the testing location due to its higher (vs forearm) sweat gland density possession.^[^ ^36^ ^]^ We investigated five EG‐treated hydrogel variants due to their wide usage in epidermal biomedical applications: a) pure polyacrylamide (PAAm), b) porous polyvinyl alcohol (PVA), c) a PVA‐PAAm blend, d) PAAm‐polyacrylic acid (PAA) blend, and e) PVA‐PAA blend. The hydrogel‐based sweat extraction does not cause any adverse effect on skin upon interfacing (Figure S2, Supporting Information). We initially observed that the osmotic sweat‐extraction rate from the fingertip can range ≈75—400 nL min^−1^ (Figure S2, Supporting Information). Moreover, a hydrogel that tends to retain more water (high swelling), allows lesser dye flow on the paper channel (Figure 2b). Such hydrogels are not suitable for extracting biomarkers whose amount in sweat is low (sub millimolar or less), as most of them would then stay trapped inside the hydrogel and go undetected in the channel. A low swelling hydrogel facilitates the highest dye flow on paper, while a deswelling hydrogel dilutes the dye on paper. All hydrogels containing PAAm swelled, with PAAm‐PAA and PVA‐PAAm gel showing the highest and least swelling, respectively (Figure 2c i). This shows that PAA and PVA enhance and restrict the swelling of the PAAm gel, respectively. The swelling nature of the hydrogels also proves that they do not cause any dilution in the channel. Except for PVA‐PAAm, the two other PVA comprising gels de‐swelled by losing water onto the paper channel. This can be attributed to the high PVA content (≈25–35%) in both these gels. Thus, both PAAm and PAA favor hydrogel swelling, while PVA restricts it. Similar experiments were also conducted with PBS‐treated hydrogels to evaluate the performance under natural perspiration (Figure 2c ii). PBS was used as the osmolyte in this case since its isotonic with sweat.^[^ ^30^ ^]^ All PBS‐treated hydrogels were de‐swelled, clearly confirming their incapability toward extracting sweat. Such understanding regarding each hydrogel contribution can strategically guide their usage at different body locations for enhanced sweat collection. Overall, the results prove that both PVA‐PAAm and pure PAAm can potentially be utilized for continuous sweat glucose sampling from the fingertip. We also evaluated the dye flow rate on the rectangular section of the paper channel for each hydrogel variant (Figure 2d i). The PVA‐PAAM showed the highest dye flow rate of ≈300 nL min^−1^, mostly since it swelled the least. Both pure PAAm and PVA‐PAA gels showed dye flow rates in the range of ≈100–200 nL min^−1^. Since PVA‐PAA hydrogel undergoes deswelling, the progression of dye on paper is guided by the inherent content of the gel and not sweat. The same reasoning holds also for pure PVA and PVA‐PAA hydrogels, where such hydrogels can dilute the sweat biomarkers. Overall, both pure PAAm and PVA‐PAAm hydrogels were the best candidates to conduct osmotic‐based sweat extraction from the fingertip. Amongst these two, the PVA‐PAAm hydrogel showed greater dye collection on paper, which was also the highest amongst all other hydrogel variants (Figures S3 and S4, Supporting Information). All PBS‐treated hydrogels showed lower dye flow (≈50 nL min^−1^, Figure 2d ii,e) in comparison to EG treated gels, due to their inability to extract enough sweat to drive the dye flow on paper (Figure S5, Supporting Information). Thus, the addition of osmosis facilitates greater sweat collection on paper as compared to natural perspiration alone. Moreover, the osmotic sweat extraction method generates ≈an 83% lower flow rate^[^ ^40^ ^]^ and ≈5–6 times higher flux inflow^[^ ^37^ ^]^ (Note S2, Supporting Information) compared to conventional iontophoretic and natural perspiration‐based sweat sampling techniques, respectively. This makes this sweat extraction technique a unique approach for greater sample collection by being less prone to dilution effects from excessive sweating. We also evaluated the osmotic contributions from both PAAm and PVA‐PAAm gels on the forearm (Figure 2f i,ii). PAAm hydrogel showed lower swelling and dye flow in comparison to the fingertip, owing to lower sweat sampling from significantly lesser sweat gland density. The PVA‐PAAM hydrogel underwent deswelling and resulted in a higher dye flow rate from the inherent EG content in the gel (Figure S6, Supporting Information). The results proved the osmotic effects to be valid for pure PAAm hydrogel and it to be better suited for continuous sweat sampling on the forearm. Furthermore, PAAm gel possessing a higher compressive modulus (Figure S7, Supporting Information) and osmotic pressure than PVA‐PAAm (Note S3, Supporting Information), justifies its usage on the forearm (offers greater pumping activity with high rigidity at lesser sweat gland sites), due to greater swelling on the fingertip (as in Figure 2c i). Overall, the estimated sweat rates on the fingertip and forearm from osmotic extraction do not aggravate glucose dilution during its transport from the sweat gland to the skin (reported through previous mathematical models and Note S2, Supporting Information),^[^ ^41^ ^]^ further justifying its usage toward SCGM development.

### 2.2 Continuous On‐body Glucose Monitoring at Rest

#### 2.2.1 Fingertip

**Figure 3:**
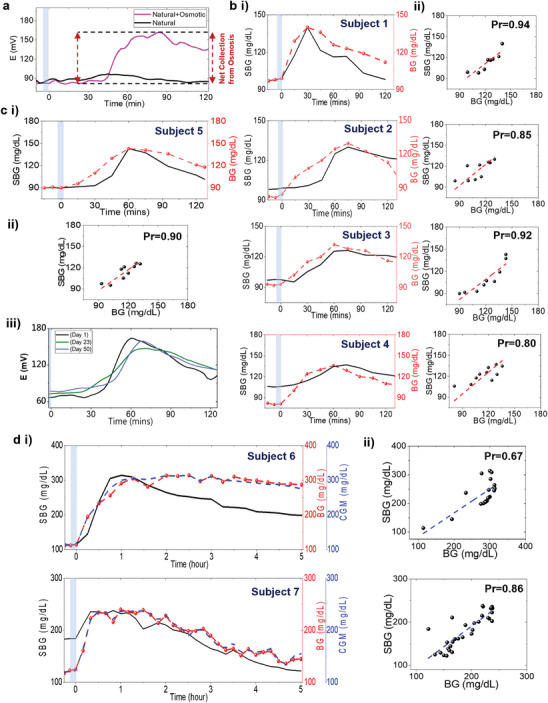
Sweat glucose trends from the fingertip. Comparative analysis of *E*(*t*) with and without the effect of osmosis after carbohydrate intake. **b i** Plots comparing the trends of *SBG* (*t*) versus *BG* (*t*) in four healthy subjects for 2 h after meal intake. The subjects fasted overnight before testing. **ii** Correlation plots between *SBG* (*t*) versus *BG* (*t*) for the same four subjects. **c i** Plot showing *SBG* (*t*) versus *BG* (*t*) trend, **ii** the correlation plot, and **iii** the stability of the glucose profile over ≈50 days for a subject. As *SBG* (*t*) showed an onset lag, repeated *E* (*t*) measurements were conducted on three different days (days 1, 23, and 50) with the same carbohydrate intake amount to check the *E*(*t*) profile stability. Similar *E* (*t*) versus *t* trends verified the subject‐specific occurrence of the sweat glucose profile. **d** Data from 2 diabetic subjects showing the **i** *SBG* (*t*) versus *BG* (*t*) trends and **ii** correlation plots after 5 h testing. The diabetic subjects fasted overnight before the test and took a prescribed fixed amount of insulin prior to meal consumption. Personalized parameters were evaluated from BG values. Similar *SBG* (*t*), *BG*(*t*), and CGM trends justify the model to be applicable to diabetic subjects as well. Blue zone: Meal intake duration.

The fingertip has been a pivotal site for conducting discrete sweat glucose measurements.^[^ ^34^, ^35^, ^37^, ^42^ ^]^ Compared to natural perspiration, osmosis facilitates a greater and continuous inflow of sweat glucose in the channel (**Figure** **3**a). The platform was tested on five healthy and three diabetic subjects, where based on the meal and physiological status, *E*(*t*) started increasing ≈20–40 min (95% CI) after the onset of BG rise, post meal intake in healthy subjects. The overall *E*(*t*) and BG concentration change ranged ≈ 45–70 mV and 40–45 mg dL^−1^ (95% CI), respectively, with peak timelines at ≈15–20 min apart (Figure S8, Supporting Information). This blood‐to‐sweat (BS) lag timeline range matches with other sweat glucose reports and is greater than the blood‐to‐ISF (BI) average lag time (≈5–10 min),^[^ ^43^, ^44^, ^45^ ^]^ reflecting the closer proximity of ISF to the blood capillaries.^[^ ^34^, ^35^, ^41^ ^]^ Furthermore, finite element analysis simulations show that a) sweat glucose covers the rectangular section of the paper channel within 10 min at the fingertip flow rate, proving that paper does not affect the glucose transport, and b) convection and advection effects play a dominant role in determining the glucose flux distribution on the channel only at higher flow rates (> 1 µl min^−1^) and high concentrations (> 1 mm), eventually demanding sweat rate based corrections (Figure S9 and Video S1, Supporting Information). All *E*(*t*) data points showed a poor correlation (Pearsons's coefficient (Pr) versus BG = 0.60), proving that it does not generate accurate information about the BG dynamics (Figure S10, Supporting Information). Such poor correlation from unprocessed sweat data have been reported previously for both passive and iontophoretically extracted sweat, which are mostly an outcome of the glucose partitioning mechanism from blood to sweat.^[^ ^22^, ^34^ ^]^ Since BG profiles are individualized, personalized calibration models are needed to convert *E*(*t*) into a concentration equivalent, so that its correlation could be studied versus BG concentration.^[^ ^34^ ^]^ *E*(*t*) was thus processed using a personalized two‐point calibration model (Note S4, Supporting Information) and converted to an equivalent SBG concentration for every subject using the following equation:

(1) $$SBG\;\;\left(t\right)=\;\left(\frac{E\left(t\right)-E_{o}+\Delta E_{o}}{\Delta E_{max}}\right)\left(BG_{max}-BG_{f}\right)$$

where *E*(*t*) is the measured potential at each time stamp, *E_o_* is the fasting potential, Δ*E_o_* is the y‐intercept, Δ*E_max_* is the maximum personalized potential post‐meal consumption, *BG_max_* is the maximum blood glucose concentration, and *BG_f_* is the fasting blood glucose concentration. Equation (1) matches a straight‐line equation of the form:

(2) $$y\left(t\right)\;=\;mx\left(t\right)+c$$

with *y* (*t*) =  *E*(*t*) − *E_o_*, $m\;=\frac{\Delta E_{max}}{BG_{max}-BG_{f}}$, *x* (*t*) =  *SBG*(*t*), and *c*  =   − Δ*E_o_*. The model uses the experimental *E*(*t*) and BG data to generate and correct the personalized parameters (*m* and *c*) for *SBG* (*t*) estimation in each subject (Figure S11, Supporting Information). The average *m* and *c* values ranged $1.34\frac{mV}{mg/dL}$ and − 138 *mV*, respectively in healthy subjects. Multiple evaluations of these parameters determine the long‐term stability of the *SBG* (*t*) profile in each subject. Overall, *SBG* (*t*) offers a direct linkage between the SCGM potential data and BG dynamics, to which the users can closely relate. A personalized *SBG* (*t*) was derived for every subject (Figure 3b,c i), which followed a similar trend as *E*(*t*) and showed an improved correlation (Pr = 0.84‐0.92) versus *BG* (*t*) from all subjects (Figure 3b,c ii). This shows that estimating *SBG* (*t*) can help understanding the BG dynamics.

Furthermore, Figure 3b,c i, and Figure S8 (Supporting Information) show delayed (by ≈30–40 min) onset of *SBG* (*t*) response in subjects 2 and 5, while 5–15 min in subjects 1,3, and 5. The onset time of *BG* (*t*) change was same for all subjects. Hence, for validation we: a) investigated the effect of sweat rate on *SBG* (*t*) (Figures S12 and S13, Supporting Information) and b) conducted repeated on‐body trials on different days to check the stability of the personalized parameter values (*m* and *c*) and calibration (Figure S14, Supporting Information). The design of the sweat rate monitoring platform was inspired from a previous work.^[^ ^46^ ^]^ Similar glucose profiles (both *E*(*t*) and *SBG* (*t*)) and similar sweat rate trends over multiple days (up to ≈2–3 months) in subjects 1, 2, and 5, verified the following aspects: a) calibration stayed valid b) every glucose profile and its onset timeline is a physiological outcome of the subject and c) osmotic based extraction can minimize the sweat rate effects on the *SBG* (*t*) profile. A typical example is demonstrated for subject 5 in Figure 3c iii, showing similar *E*(*t*) profile till 50 days. All these factors can collectively contribute toward finalizing the personalized parameter values for each subject and justify their future usage to obtain a blood calibration‐free *SBG*(*t*) profile for all future tests. The subject remeasured *SBG* (*t*) on the 65^th^ day (since first calibration) by using the fixed personalized parameter values (till day 50) and found the profile to be similar (to both BG and trends till day 50). This means that subject 5 does not necessarily need to repeat the BG calibration till 65 days (after calibrating on day 1). Here we checked the long‐term stability of the calibration till 50 days, but ideally, the subject can even fix these parameters with 2–3 repeated measurements consecutively. Sporadic *BG*(*t*) measurements can certainly help in updating these values and checking the calibration stability, especially for diabetic subjects, since reports support the reduction of health consequences with improved lifestyle (like diet, BMI, and body composition).^[^ ^46^, ^47^, ^48^ ^]^ All these metrics can be stored/analyzed by a mobile application when targeting toward building a device. The SCGM was also tested on three T1‐D subjects for 5 h along with a commercial CGM (Figure 3d i). All subjects showed rapid onset of glucose increment in the channel and a net change of ≈80–110 mV and 100–200 mg dL^−1^ in *E*(*t*) and *BG*(*t*), respectively, in ≈45–60 min after meal intake. Despite different personalized parameters ($m\;=\;0.64\frac{mV}{mg/dL}$ and, *c*  =   − 98.5 *mV*) versus healthy subjects due to high *BG* (*t*) values (Figure S11, Supporting Information), the decently good correlation (average Pr = 0.85) between *SBG* (*t*) and *BG* (*t*) from all subjects proved that SCGM and the calibration model could be applied on diabetic subjects as well (Figure 3d ii). Moreover, the observed BS time lag matched with the reported BS time lag of iontophoretically extracted sweat (≈10‐15 mins)^[^ ^49^ ^]^ and BI time lag. This further shows the minimal effect of passive sweat rate on the *SBG* (*t*) profile and the enhanced glucose diffusion from blood to sweat at the fingertip in diabetic subjects.

#### 2.2.2 Forearm

**Figure 4:**
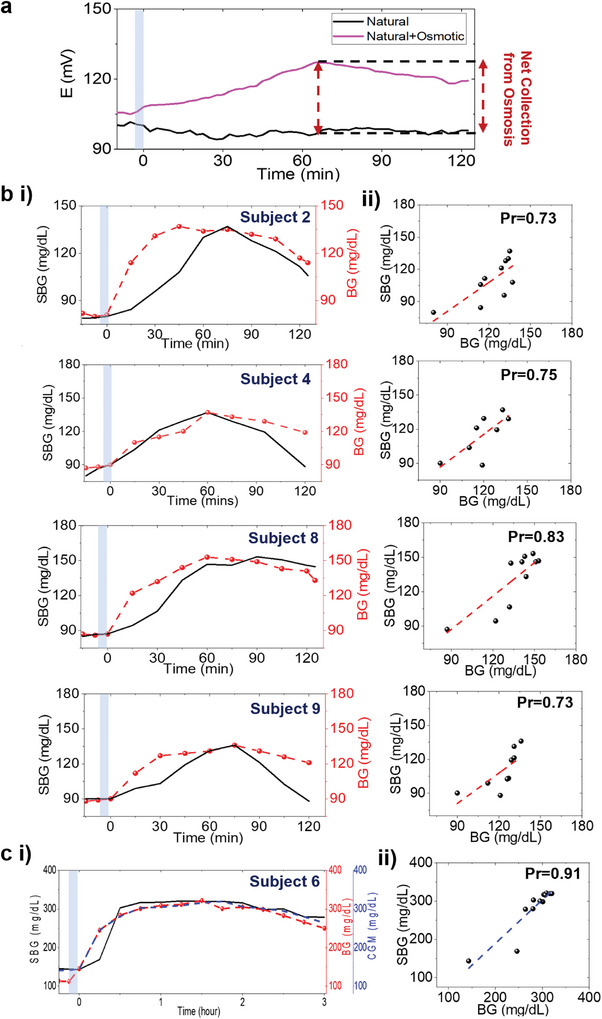
Sweat glucose trends from the forearm. a Comparative analysis of *E*(*t*) with and without the effect of osmosis after carbohydrate intake. EG and PBS‐soaked hydrogels were used to track osmotic and non‐osmotic effects, respectively. **b i** Plots comparing the trends of *SBG* (*t*) versus *BG* (*t*) over for four healthy subjects after overnight fasting. **ii** Correlation plots between *SBG* (*t*) versus *BG* (*t*) for the same four subjects. **c i** Plot showing the *SBG* (*t*) versus *BG* (*t*) trend and **ii** their correlation for one diabetic subject. The same protocol as the fingertip test was followed by this diabetic subject. Personalized parameters were evaluated from BG values. Blue zone: Meal intake duration. Other conditions, as in Figure 3.

The SCGM was also tested on the forearm since it is a more convenient location (than fingertip) for interfacing wearable patches. **Figure** **4**a shows how osmosis leads to even greater glucose extraction over time on the forearm (vs natural perspiration), post meal consumption. All healthy subjects showed a net *E*(*t*) change of ≈5–15 mV (95% CI), which peaked ≈75—90 min after meal consumption and showed a lag‐time ≈15—45 min (Figure S15, Supporting Information). The overall lower change in *E*(*t*) and greater time delay (than fingertip) toward approaching *SBG_max_* (*t*) arises from the slow reaction kinetics of glucose in the channel, which can be attributed to the lower sweat flow rate due to lower sweat gland density in the forearm (Figure S16, Supporting Information).^[^ ^36^ ^]^ These factors also lead to low average values ($m\;=\;0.20\;\frac{mV}{mg/dL}$ and *c*  =   − 17 *mV*) of the personalized parameters and *SBG* (*t*) in healthy subjects (Figure 4a,b i; Figure S17, Supporting Information). Even with a low sweat rate, the *SBG*(*t*) profile from subjects 2 and 4 did not possess a delayed onset, unlike the fingertip. This shows the importance of the testing location and further justifies how personalized factors play a key role toward determining the overall *SBG* (*t*) profile. The diabetic subjects showed a net *E*(*t*) change of ≈50–60 mV after 1 h post meal consumption, and both *E*(*t*) and *SBG* (*t*) profiles possessed a relatively slower (vs fingertip) decay rate (Figure 4c). However, *E*(*t*) showed a poor correlation (Pr = 0.30) versus *BG* (*t*) (Figure S18, Supporting Information), proving that the forearm is not a suitable location for measuring sweat glucose levels at rest. The estimated *SBG* (*t*) from Equation (1). showed an improved and reasonable correlation (Pr = 0.72‐0.80) versus *BG* (*t*), proving that the calibration model could still be used on the forearm to estimate BG dynamics (Figure 4b ii). The diabetic subject showed a rapid increase in the CGM and *SBG*(*t*) readings (Figure 4c **i**). The personalized values ranged almost similar $(m\;=\;0.36\;\frac{mV}{mg/dL}$ and *c*  =   − 46 *mV*) versus healthy subjects and a good correlation (Pr = 0.88) between *SBG*(*t*) versus *BG*(*t*) was observed for this subject (Figure 4c **ii**). Overall, a lower (vs fingertip) *m* for forearm from both healthy and diabetic subjects supports lesser extracted glucose, which reflects lower sweat gland densities.

### 2.3 Extended Validation and Analysis of SCGM

**Figure 5:**
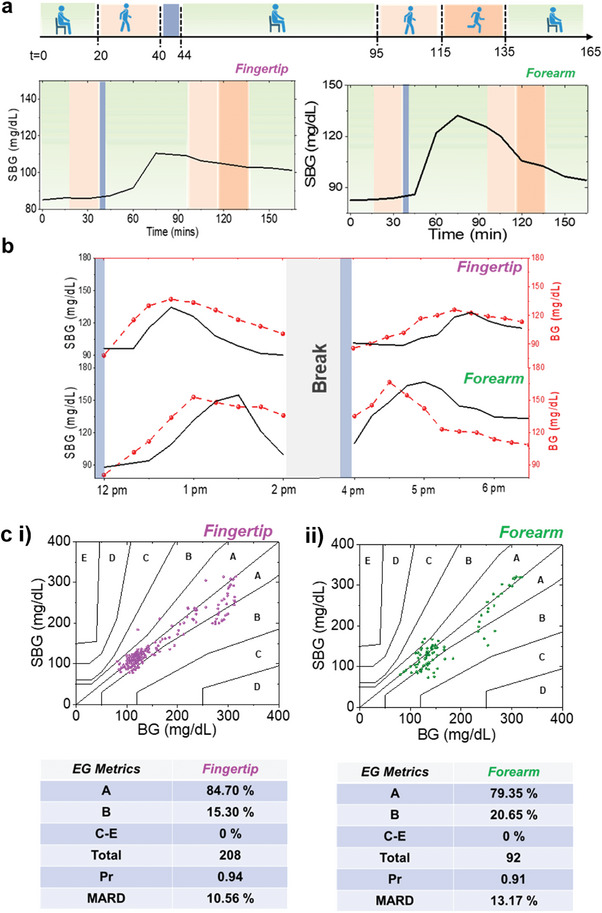
Long‐term analysis of SCGM. a *SBG*(*t*) versus *t* profiles of subject 2 under varying physiological activities from fingertip and forearm during diverse daily activities: Green: Rest, Blue: Meal intake duration, Light red: Medium intensity walking, Dark red: High intensity walking. The subject had fasted overnight before testing. $m\;=\;2.25\;\frac{mV}{mg/dL},c\;=\;-132\;mV$ and $m\;=\;0.32\;\frac{mV}{mg/dL},c\;=\;-25\;mV$ were used as personalized parameters for fingertip and forearm, respectively. **b** *SBG* (*t*) versus *t* profiles for fingertip and forearm with multiple meals by wearing a single patch. During the break, the subject continuously wore the patch and did not perform any high exertion activity. **c** Consensus error grid plots and analysis from all data points obtained from the **i** fingertip and **ii** forearm trials.

The extended operational validity of the SCGM was tested under two conditions: a) daily routine activities and b) multiple meals. **Figure** **5**a shows the *SBG*(*t*) trends from subject 2 undergoing multiple rounds of routine activities with varying physiological conditions such as sitting, and medium and high‐intensity outdoor walking. *SBG*(*t*) was derived using personalized calibration parameters. To prevent the interference of active sweat, the tests were not conducted under high exertion activities (like exercise, and running). The subject underwent overnight fasting and wore the SCGM the next day for 20 min at rest, and then during a medium‐intensity outdoor walk for 20 min. *SBG*(*t*) from the fingertip and forearm did not change during this time. Negligible variations in sweat rate were even observed during the outdoor walk, confirming its negligible effect on *E*(*t*) (Figure S19, Supporting Information). This is also evident from the in vitro sweat rate studies, where significant *E*(*t*) changes were observed only under high flow rate (> 1 µL min^−1^) and concentration (> 1 mM) (Figure S9, Supporting Information). The subject then took a meal and rested for the next 60 min. The onset of *E*(*t*) increase was after 15—20 min and a net *E*(*t*) change of ≈45 mV was observed after 45—50 min on the fingertip. The *SBG*(*t*) increased by ≈25 mg dL^−1^. All these timelines and values were in range with the trends of subject 2 in Figure 3b i. *E*(*t*) and *SBG*(*t*) changed by ≈15 mV and ≈45 mg dL^−1^ on the forearm after ≈35–40 min, post meal. The lesser increment of *E*(*t*) and its faster drop can be an outcome from the lesser amount of extracted glucose due to lower sweat gland density on the forearm. No change in *E*(*t*) was observed without meal, proving that the signal was from sweat glucose (Figure S20, Supporting Information). After *E*(*t*) started to drop, the subject started another round of medium intensity outdoor walk and then a high intensity outdoor walk, each for 20 min. Such a protocol was followed to let the natural drop of *E*(*t*) (due to glucose) stay minimally impacted from the sweat rate effects. The *E*(*t*) profile from the fingertip underwent a continuous decay, but the sweat rate increased by ≈7–8 nl min^−1^ cm^−2^ during this walking period (Figure S19, Supporting Information). However, the constant decay rate of *E*(*t*) profile (post peak appearance) justified the negligible effect from sweat rate. The forearm sweat rate increased by ≈2 nl min^−1 ^cm^−2^ during walking, but no significant change was observed in its *E*(*t*) trend. The subject was then allowed to rest for the next 30 min and no changes in *E*(*t*) and sweat rate were observed. All these results were further supported by the *E*(*t*) trends obtained from outdoor walking test right after the meal intake (Figure S21, Supporting Information). Similar decay onset timeline and rate of *E*(*t*) revalidated the minimal influence of outdoor walking on the glucose profile. Furthermore, greater sweat rate variations were observed under natural perspiration (with 0.1 PBS hydrogel) during outdoor testing (Figure S19, Supporting Information). However, the sweat rate varied ≈from 10 nl min^−1^ cm^−2^ before a meal and ≈40–45 nl min^−1 ^cm^−2^ after the meal at rest on the fingertip, justifying the necessity of performing sweat rate‐based corrections for accurate *SBG* (*t*) estimation. Lesser fluctuations (≈2 nl min^−1^cm^−1^‐ EG hydrogel and ≈5 nl min^−1 ^cm^−2^‐ PBS hydrogel) were observed on the forearm due to its inherent low sweat generation rate. Hence, the osmotic effect helps in reducing the sweat rate variations under varying physiological conditions.

The SCGM was also tested at rest on a subject with two consecutive similar meal intakes (Figure 5b). Both *BG* (*t*) and *SBG* (*t*) increased by ≈50 and ≈40 mg dL^−1^, respectively, within ≈45 min on the fingertip after the first meal. The second meal was consumed after 2 h, where the net *BG* (*t*) value peaked by ≈45 mg dL^−1^ after ≈105 min. The BS onset and peak timeline ranged ≈15–30 and ≈5–10 min after both meals. The delayed peaking of blood glucose can be an outcome of subject specific glucose metabolism in the body from multiple meals. To validate this, we performed the test also on a new subject (Figure S22, Supporting Information). Here, the BS onset time lag for both meals were similar, but BS peak time lag ranged ≈from 15—30 min, and less than 5 min after the first and second meal intake, respectively, eventually proving it to be a subject‐specific output. The *SBG* (*t*) changed by ≈30 mg dL^−1^ after the second meal intake in Figure 5b, proving that multiple meals can affect the timeline of peak glucose appearance. The BS onset time lag ranged ≈between 15 min and ≈45 min from meals 1 and 2, respectively, while the BS peak time lag was found to be within 15 min. Overall, the mean absolute relative difference (MARD) between *SBG* (*t*) and *BG* (*t*) before and after second meal ranged ≈18.30% and 14%, respectively. The same test was also conducted on the forearm where both *BG* (*t*) and *SBG* (*t*)increased by ≈65 and ≈60 mg dL^−1^, respectively after the first meal. The BS peak time lag ranged ≈30 min with no onset time lag. *BG* (*t*) increased by ≈30 mg dL^−1^, while *SBG* (*t*) peaked by ≈55 mg dL^−1^ after the second meal with no change in the BS peak time lag. The MARD before and after second meal was ≈13% and 16.50%, respectively.

The acquired *SBG* (*t*) data points from the fingertip and forearm sweat were plotted in two separate T1‐D consensus error grid (CEG) plots to evaluate the accuracy of *SBG* (*t*) versus *BG* (*t*).^[^ ^50^ ^]^ All fingertip (n = 208, MARD = 10.56%) and forearm (n = 92, MARD = 13.17%) data points lied in the A+B zone of CEG confirming that none of the data points drastically affected the outcomes of the trials (Figure 5c i,ii). The fingertip data points showed less scattering versus forearm data points in healthy subjects (BG ≈80–160 mg dL^−1^), while the scattering slightly increased at higher BG values in Figure 5c i mainly due to subject 6 and subject 10 (Figure S8, Supporting Information). These observations were further supported by the 95% CI plots and the Clarkes error grid plot. (Figures S23 and S24, Supporting Information). Furthermore, greater accuracy (lower MARD), versus *BG* (*t*)) of *SBG* (*t*), was observed when *BG* (*t*) change per unit time ranged −1 to 1 mg dL^−1^ min^−1^ in healthy subjects. For diabetic subjects, MARD stayed less than 15% throughout −2 to 2 mg dL^−1^ min^−1^. (Figure S25, Supporting Information). An overall Pr > 0.90 justified that estimated *SBG* (*t*) reading from both fingertip and forearm with our SCGM can accurately represent the BG dynamics in the body. Since MARD from fingertip was lower than the forearm, it proved it to be a better location for conducting real‐time BG analysis with passive perspiration.

## 3 Conclusion and Outlook

We report here a passive perspiration‐based continuous sweat glucose monitoring system, which can fully operate at rest without requiring any on‐skin sweat stimulation prior measurement and can deliver dynamic BG information. We found that by enabling osmotic pressure difference (vs sweat) through a hydrogel on‐skin and by using a paper microfluidic channel as the transport media, continuous and extended sweat glucose collection is possible. We even use a self‐powered potentiometric technique for glucose estimation, which makes our system completely independent of external power for running simultaneously both sweat extraction and sensing operations and be compatible for integration with low‐power electronic systems. The output potentiometric signal, blood glucose data, and sweat rate variations have been collectively utilized to develop and validate personalized calibration models for each subject, through which the BG levels (*SBG* (*t*)) can be predicted. The long‐term stability of the calibration parameters determines the *SBG* (*t*) accuracy versus measured BG concentration. Such models even improved the overall correlation between *SBG* (*t*) and *BG* (*t*), proving the necessity of addressing personalized variations in each subject. Moreover, osmotically withdrawn sweat rate was seen to have minimal effects on the overall *SBG* (*t*) profile under varying physiological conditions. *SBG* (*t*) from the fingertip sweat even possessed a lower MARD, supporting it to be a better location for glucose monitoring. Furthermore, from the practicality point of view and valuing users’ comfort, the SCGM could even be used on the forearm due to the good correlation between *SBG* (*t*) and *BG* (*t*).

Our attempt here was to investigate the potential of passively harvested sweat (in parallel to ISF) as an alternative biofluid candidate for acquiring real‐time BG dynamics in the body – a concept which has not been evaluated before. The study was conducted in a small cohort to examine the potential of our SCGM toward generating accurate dynamic BG information during daily life activities. However, future studies will require rigorous large‐scale clinical trials to further understand the performance of the system. We believe that the accuracy of every SCGM will depend on the following factors: a) human physiology guiding the partitioning of glucose from blood to sweat, b) sweat extraction mechanism, c) dilution from sweat rate variations, d) microfluidic channel effects, e) sensor performance, and f) calibration protocols. These points have been highlighted in recent reports.^[^ ^51^, ^52^ ^]^ We have tried to minimize certain effects (b–f) in our SCGM, so that it can only capture the physiological aspects. Our results also suggest that the overall BS lag time comprises an onset lag time and a peak appearance lag time, in which the former depends more on the static physiological status (like fasting effects on BG or pre‐existing health conditions) of the subject, while the latter on the dynamic physiology (real‐time glucose metabolism) and system aspects (like transport effects and glucose detection mechanism). Hence, every sweat glucose researcher should target addressing these issues on a fundamental and personalized level to develop an accurate SCGM. Iontophoretically extracted sweat from forearm has previously shown a BS time lag of ≈10 min with a ≈23% MARD in diabetic subjects, while passive sweating has shown BS time lag up to an hour.^[^ ^49^, ^50^, ^53^ ^]^ Such a high MARD can result from dilutions caused by the high sweat volumes, while a high lag time can result from improper and uncontrolled sweat sampling. This demands further scope of improvement in system development for BG estimation with greater accuracy, which our platform has attempted to address (diabetic average MARD ≈13.2%, n = 98). The reasons for the BS lag time are related to that of BI (due to vascularization of sweat glands),^[^ ^29^ ^]^ which include factors like physiological induced time delay, delay of glucose transport from blood to ISF, tissue perfusion, and signal processing delays.^[^ ^41^, ^43^, ^44^, ^45^, ^54^ ^]^ However, unlike ISF, the glucose amount in epidermal sweat can be additionally affected by the sweat extraction mechanism, which can govern the skin layer metabolic uptake, dermal clearance rate of glucose, and epithelial glucose permeability.^[^ ^29^, ^38^, ^41^ ^]^ All these factors will govern how much glucose is available on skin‐surface and can collectively contribute to the overall BS time lag. Hence, the causes of such lag time in every platform should be first investigated cases by case before attempting to correct it using algorithms, as used widely by ISF‐based CGMs.^[^ ^55^, ^56^, ^57^, ^58^ ^]^ Such algorithms aim to exclude noisy signals during calibration, correct BG time lags, and point out artifacts from sensor degradation to improve the overall MARD, but can even worsen it if not executed properly.^[^ ^55^, ^56^, ^57^, ^58^ ^]^ Studies have shown that even the ISF glucose profiles and time lags follow complex mechanisms (compared to BG) which require more than just a time‐shift correction, further proving the potential complexities associated with sweat glucose dynamics.^[^ ^59^ ^]^ Moving forward, we know that commercial ISF based CGMs are already in widespread use due to their usage conveniency, robust performance (≈10‐14 days operation time with <5 mins BI lag time), and accuracy (MARD≈7–8%).^[^ ^8^, ^58^ ^]^ This certainly calls the need for further SCGM development and additional investigation since sweat is relatively more accessible on‐skin and can generate steady secretion rates (greater available volume) versus ISF. The successful execution of such passive SCGMs can surely prove to be a great addition toward the development of next generation self‐administered glucose monitoring devices.

## 4 Experimental Section

### Hydrogel Preparation

The hydrogel was prepared in the following ways:

- Pure polyacrylamide (PAAm) gel: The hydrogel solution comprised acrylamide, N,N′‐methylenebisacrylamide (cross‐linker), 2‐hydroxy‐4′‐(2‐hydroxyethoxy)−2‐methylpropiophenone (photoinitiator) in DI water in the mass ratio of 0.22:0.0046:0.0015:0.7740.
- Pure polyvinyl alcohol (PVA) gel: A PVA (MW≈30 000) solution (0.1 g ml^−1^), KOH solution (0.2 g ml^−1^), and a sucrose solution (1.3 g ml^−1^) were initially prepared. Then all components were mixed in the mass ratio of 0.38:0.526:0.097 in a petri dish (9 cm diameter) and vacuum‐dried overnight. The gel was then washed with 0.1 M PBS multiple times until the pH reached ≈7.4.
- PVA‐PAAm gel: The hydrogel solution comprised acrylamide, N,N′‐methylenebisacrylamide (cross‐linker), 2‐hydroxy‐4′‐(2‐hydroxyethoxy)−2‐methylpropiophenone (photoinitiator), DI water, and 0.1 g ml^−1^ PVA solution in the mass ratio of 0.0293:0.006:0.0017:0.8375:0.1256.
- PAAm‐polyacrylic acid hydrogel (PAA): The hydrogel solution comprised acrylamide, N,N′‐methylenebisacrylamide (cross‐linker), 2‐hydroxy‐4′‐(2‐hydroxyethoxy)−2‐methylpropiophenone (photoinitiator), DI water, and 1 g ml^−1^ polyacrylic acid (Mw≈450000) in the mass ratio of 0.22:0.0046:0.015:0.7740:0.01
- PVA‐PAA hydrogel: The PVA‐PAA hydrogel is prepared by the same method as pure PVA with 1 wt% polyacrylic acid (PAA). A PVA solution (0.1 g ml^−1^) and a PAA solution (0.001 g ml^−1^) were prepared together. A KOH solution (0.2 g ml^−1^) and sucrose (1.3 g ml^−1^) are prepared and mixed with solution 1.
- All gel solutions were poured onto a petri dish and cured under UV light (36 W) for an hour to get the gel. The gel was then soaked in pure ethylene glycol (EG) overnight. Disks of 6 mm diameter were then punched out and stored in a vial of fresh EG for future use.

### Fabrication of Electrodes–Glucose sensor

The sensor comprises a two‐electrode system developed on a SEBS sheet. SEBS G1645 (40% solution in toluene) was used. Initially, two current collector lines were printed using Ag/AgCl ink and cured in the oven at 80 °C for 20 min. The anode (4 mm x 1 mm) was prepared using carbon ink. The carbon consists of graphite, super P conductive carbon black, SEBS (G‐1645 40 wt % in toluene), and toluene 6:1:8.4:2.1 weight ratios. The ink was screen printed on one end of a current collector line and cured at 80 °C for 20 min. The cathode (4 mm x 1 mm) was prepared on one end of the other current collector line by screen printing subsequent layers of carbon and Ag_2_O ink and then cured at 80 °C for 20 min. The Ag_2_O ink was prepared by mixing Ag_2_O, Super Porous carbon black, and GBR binder (21 wt% in acetone) in a 1.900:0.100:3.166 weight ratio. All inks were mixed in a plenary mixer at 2500 rpm for 10 min or until homogeneous before repeated usage. The junction between the current collector and the printed electrodes was insulated before testing.

### Fabrication of Electrodes–Sweat rate sensor

The sweat rate sensor comprised of two screen‐printed carbon electrodes (1 mm x 30 mm) on a SEBS sheet. The ink was prepared by mixing the carbon ink with 1 wt% Triton‐X‐100. Current collector lines using Ag/AgCl ink were also printed at the end of both electrodes.

### Potentiometric Glucose Sensor Preparation and Characterization

The glucose sensor was developed by subsequently drop casting 5, 5, 1, and 1 µL of 0.2 m NQ solution (solvent comprised of 2 mg mL^−1^ MWCNT‐CCOH in 1:9 ethanol to acetone), 40 mg mL^−1^ glucose oxidase (GOx) solution, 1% glutaraldehyde in DI water, and 1% chitosan in 0.1 m acetic acid, respectively. The sensor was preserved overnight (≈4 °C) before usage. Chronoamperometric evaluation was conducted at −0.1 V versus Ag/AgCl. For potentiometric detection, linear sweep voltammetry (LSV) was initially performed at 1 mV sec^−1^ to finalize the external load for maximum signal resolution. An external resistor of 1MΩ (obtained from LSV) was always soldered between the two electrodes as the discharge load during all in vitro and on‐body studies. On‐body EIS validations for the glucose sensor were conducted at E_dc_ = −0.05 V versus Ag/AgCl, E_ac_ = 10 mV, and set frequency = 50 KHz. The sweat rate sensor was operated at: E_dc_ = 0 V, E_ac_ = 10 mV, and set frequency = 100 KHz. All data were acquired using potentiostats from Autolab (PGSTAT101) and PalmSense4.

### SCGM Fabrication

The SCGM comprised four major components: SEBS sheet, hydrogel, electrochemical glucose biosensor, and a paper microfluidic channel (Whatman grade #1). All components were sandwiched between two SEBS sheets of 65 mm x 2.1 mm x 1.5 mm. A circular hole of 6 mm diameter was punched out at one end of the bottom sheet to host the hydrogel disk. The serpentine design of the paper channel was cut out using a CO_2_ laser cutter, such that its circular inlet section stayed sandwiched between the hydrogel and skin, while the sensor (cathode facing inlet) was orthogonally interfaced to the thin rectangular section of the paper channel. The sweat rate sensor was interfaced to the paper channel as shown in Figure S12 (Supporting Information).

### Hydrogel Performance On‐skin

All hydrogels were tested on the fingertip to estimate their sweat withdrawing capacity via osmosis. A small patch like the SCGM (without the biosensor and large area paper channel) design was separately prepared using a paper channel (circular end: 6 mm diameter, width: 2 mm, and length 40 mm) and tested with the different hydrogel variants. The fingertip was initially stained with a red‐colored food dye solution (prepared in ethanol) and the circular end of the channel with the hydrogel on top was interfaced over it. The wicking of the dye solution on the paper channel was analyzed through image analysis to determine the sampled dye intensity and dye velocity, and eventually the hydrogel performance. Hydrogel weights were also measured before and after experiments to assess their performance.

### Finite Element Analysis Simulations

ANSYS Fluent was used to conduct fluid flow simulations. The fluid flow behavior is described by the conservation of momentum for incompressible flow equation with an additional term to account for the effects of porous media:

(3) $$\frac{\partial }{\partial t}\left(\rho v\right)\;+\nabla \cdot \left(\rho \mathrm{vv}\right)\;=\;-\nabla p\;+\mu \;\nabla^{2}\nu \;+S$$

where ρ,  ν,  *t*,  *p*,  μ and *S* denote liquid density, flow velocity, time, pressure, viscosity, and the additional momentum source term, respectively. μ  =  0.001 *Pa*.*s* and ρ of the fluid mixture was calculated based on volume‐mixing law, from the respective $\rho_{water}=\;1000\;\frac{kg}{m^{3}}$ and $\rho_{glucose}=\;1540\;\frac{kg}{m^{3}}$. The source term is composed of two parts: a viscous loss term and an inertial loss term.

(4) $$Si\;=\;-\left(\frac{\mu }{\alpha }v_{i}\;+\;C_{2}\rho \left|v\right|v_{i}\right)\;$$

(5) $$a=\frac{Dp^{2}\in^{3}}{150{\left(1-\in \right)}^{2}}\;\mathrm{and}\;C_{2}=\frac{3.5\left(1-\in \right)}{Dp\in^{3}}$$

where *D_p_* and ∈ denotes the mean particle diameter and porosity of Whatman Grade #1 paper, respectively, with the value of 1.1 × 10^−5^ m and 0.707.^[^ ^60^ ^]^

For a fluid mixture of glucose and water, the model predicts the local mass fraction of each species, *Y_i_*, through the solution of a convection‐diffusion equation below:

(6) $$\frac{\partial }{\partial t}\left(\rho Y_{i}\right)+\nabla \cdot \;\left(\rho v\;Y_{i}\right)=\;-\nabla \cdot J_{i}$$

(7) $$J_{i}\;=\;-\rho D_{i,m}\;\nabla Y_{i}$$

where *D*_*i*,*m*_ is the mass diffusion coefficient and *J_i_* is the flux for species *i* in the mixture. *D_glucose_* =  6 × 10^−6^ *cm* ^2^/*sec*

### On‐body Testing Protocol

All on‐body trials were conducted based on the approved IRB (#130 003, UCSD). Written consent was acquired from all participants before the test. All subjects fasted overnight before testing. The fingertip and forearm areas were initially cleaned using alcohol wipes and DI water before placing the SCGM. After placing the patch, it took ≈30—45 min for the glucose sensor to reach its stable discharge potential. Fasting blood glucose concentration was obtained using the “Accu‐Chek Guide Me” monitor through fingerpicking. The diabetic subjects had taken intravenous insulin (10‐12 units) prior to meal intake. All subjects then consumed ≈100 gm carbohydrate bolus (via meal) and stayed in rested conditions for the next 2—5 h. Blood glucose was then measured every 15 min for all subjects. CGM data was monitored in diabetic subjects. All outdoor glucose data were collected under mild‐high intensity walking using EmStat Blue 3 potentiostat. The sweat rate was measured simultaneously from the index finger/forearm of the other arm using PalmSense 4 both under rest and outdoor testing. The MARD was calculated using:

(8) $$MARD\;=\frac{1}{N}\;\sum_{i\;=\;1}^{N}\left(\frac{BG{\left(t\right)}_{i}-SBG{\left(t\right)}_{i}}{BG{\left(t\right)}_{i}}\right)\times 100$$

where, *N*: total data points, *BG*(*t*)_*i*_: measured blood glucose concentration, *SBG*(*t*)_*i*_: estimated sweat‐based blood glucose.

## Conflict of Interest

The authors declare no conflict of interest.

## Author Contributions

T.S., M.I.K., S.S.S., and L.Y. contributed equally to this work. T.S., L.Y., and J.W. conceived the idea. T.S, I.K, S.S.S, S.E, C.Z, O.D, S.S, and S.D performed experiments. T.W., J.H., and T.S. performed fluid simulations. A.A. and T.S. performed mechanical testing of the hydrogel. T.S, I.K, S.S.S, L.Y, and J.W analyzed the data. T.S, I.K, and S.S.S designed the human subject studies. T.S. and J.W. contributed to writing, reviewing, and editing. J.W. supervised the project. All authors substantially contributed to the research and reviewed the manuscript.

## Supporting information



Supporting Information

Supplementary Viedo 1

## Data Availability

The data that support the findings of this study are available from the corresponding author upon reasonable request.
