# Supplementary material for: A Passive Perspiration Inspired Wearable Platform for Continuous Glucose Monitoring
Source: Adv Sci (Weinh). 2024 Sep 12;11(41):2405518. doi: 10.1002/advs.202405518 (PMC11538657; doi:10.1002/advs.202405518)
Supplement: Supplementary file 1 — Supporting Information [file ADVS-11-2405518-s001.pdf]

ADVANCED SCIENCE
Open Access

Supporting Information

for *Adv. Sci.*, DOI 10.1002/advs.202405518

A Passive Perspiration Inspired Wearable Platform for Continuous Glucose Monitoring

*Tamoghna Saha, Muhammad Inam Khan, Samar Singh Sandhu, Lu Yin, Sara Earney, Chenyang Zhang, Omeed Djassemi, Zongnan Wang, Jintong Han, Abdulhameed Abdal, Samarth Srivatsa, Shichao Ding and Joseph Wang**

A Passive Perspiration Inspired Wearable Platform for Continuous Glucose Monitoring

Tamoghna Saha[a*], Muhammad Inam Khan[a*], Samar Singh Sandhu[a*], Lu Yin[a*], Sara Earney[a], Chenyang Zhang[a], Omeed Djassemi[a], Abdulhameed Abdal[c], Zongnan Wang[c], Jintong Han[c], Samarth Srivatsa[b], Shichao Ding[a], and Joseph Wang[a$]

[a] *Aiiso Yufeng Li Family Department of Chemical and Nanoengineering, University of California San Diego, La Jolla, CA 92093, USA*

[b]*Department of Bioengineering, University of California San Diego, La Jolla, CA 92093, USA*

[c]*Department of Mechanical Engineering, University of California San Diego, La Jolla, CA 92093, USA*

[*] *Equal Contribution*

[$] *Corresponding Author:* josephwang@ucsd.edu

Table of Contents

1. **Supplementary Note 1:** Fluid flow conditions in conventional microfluidic channels.
2. **Supplementary Figure 1:** Electrochemical characterization of glucose sensor.
3. **Supplementary Figure 2:** Hydrogel effect on fingertip and estimation of fingertip sweat rate with validation using a syringe pump.
4. **Supplementary Figure 3:** Cumulative dye intensity trend on the fingertip for all hydrogel variants over two hours.
5. **Supplementary Figure 4:** Dye penetration of ethylene glycol hydrogels (osmotic + natural) from fingertip.
6. **Supplementary Figure 5:** Dye penetration of PBS hydrogels (only natural perspiration) from fingertip,
7. **Supplementary Figure 6:** Dye penetration of EG treated pure PAAm and PVA-PAAm hydrogels from the forearm.
8. **Supplementary Note 2:** Flux analysis with osmotic sampling.
9. **Supplementary Note 3:** Theoretical estimation of hydrogel osmotic pressure.
10. **Supplementary Figure 7:** Mechanical testing and SEM images of hydrogels.
11. **Supplementary Figure 8:** Sensor data from fingertip.
12. **Supplementary Figure 9:** Finite element simulations and in-vitro study of flow rate effects.
13. **Supplementary Figure 10:** Correlation of potential data from fingertip vs. BG.
14. **Supplementary Figure 11:** Slope and intercept analysis from fingertip sweat.
15. **Supplementary Figure 12:** Sweat rate sensor integration with SCGM.
16. **Supplementary Figure 13:** Sweat rate analysis from fingertip**.**

17. **Supplementary Figure 14:** Stability analysis of personalized calibration models with fingertip sweat.

18. **Supplementary Figure 15:** Sensor data from forearm.

19. **Supplementary Figure 16:** Sweat rate analysis from forearm.

20. **Supplementary Figure 17:** Slope and intercept analysis from forearm sweat.

21. **Supplementary Figure 18:** Correlation of potential data from forearm vs. BG.

22. **Supplementary Figure 19:** Sweat rate vs E(t) analysis during outdoor activities.

23. **Supplementary Figure 20:** On-body control studies during outdoor activities.

24. **Supplementary Figure 21:** Data from instantaneous walking after meal consumption during outdoor activities.

25. **Supplementary Figure 22:** Fingertip sweat glucose profile of a subject with multiple meals.

26. **Supplementary Figure 23:** Overall analysis of $SBG\ (t)$ trends.

27. **Supplementary Figure 24:** Clarke's error grid analysis from fingertip and forearm.

28. **Supplementary Figure 25:** MARD distribution vs. rate of BG change.

29. **Supplementary Video V1:** Finite element simulation showing glucose (inlet concentration = 50 μM) flux profile on the paper channel at 300 nL/min for 10 minutes.

Supplementary Information

Supplementary Note 1: Fluid flow conditions in conventional microfluidic channels

The pressure drop in a conventional rectangular microfluidic channel is evaluated by:

$$\Delta P = \frac{12\left[1 - \frac{192H}{\pi^5 W}\ \tanh\left(\frac{\pi W}{2H}\right)\right]^{-1} \mu Q L}{W H^3}$$

where, ΔP: pressure drop across the channel, W: width of channel, H: channel height, Q: fluid flow rate, L: channel length, μ: viscosity of fluid.

For a typical rectangular microfluidic channel ($W = 1000\ \mu m, H = 100\ \mu m,$ and $L = 1\ cm$)[1] used in sweat sensing, ΔP across the channel can range from 6-20 kPa under active sweating ($Q = 1 - 3\mu L/\mathrm{min}$). The sweat gland secretory pressure ranges ~ 70 kPa under active sweating.[2] This proves that sweat will into the channel due to greater hydrostatic pressure. However, if $W = 100\ \mu m$, $\Delta P = 150\ kPa$, which shows that sweat will not enter the channel. Thus, the hydraulic resistance of a microfluidic channel is governed by its dimensions, which eventually dictates the sweat flow.

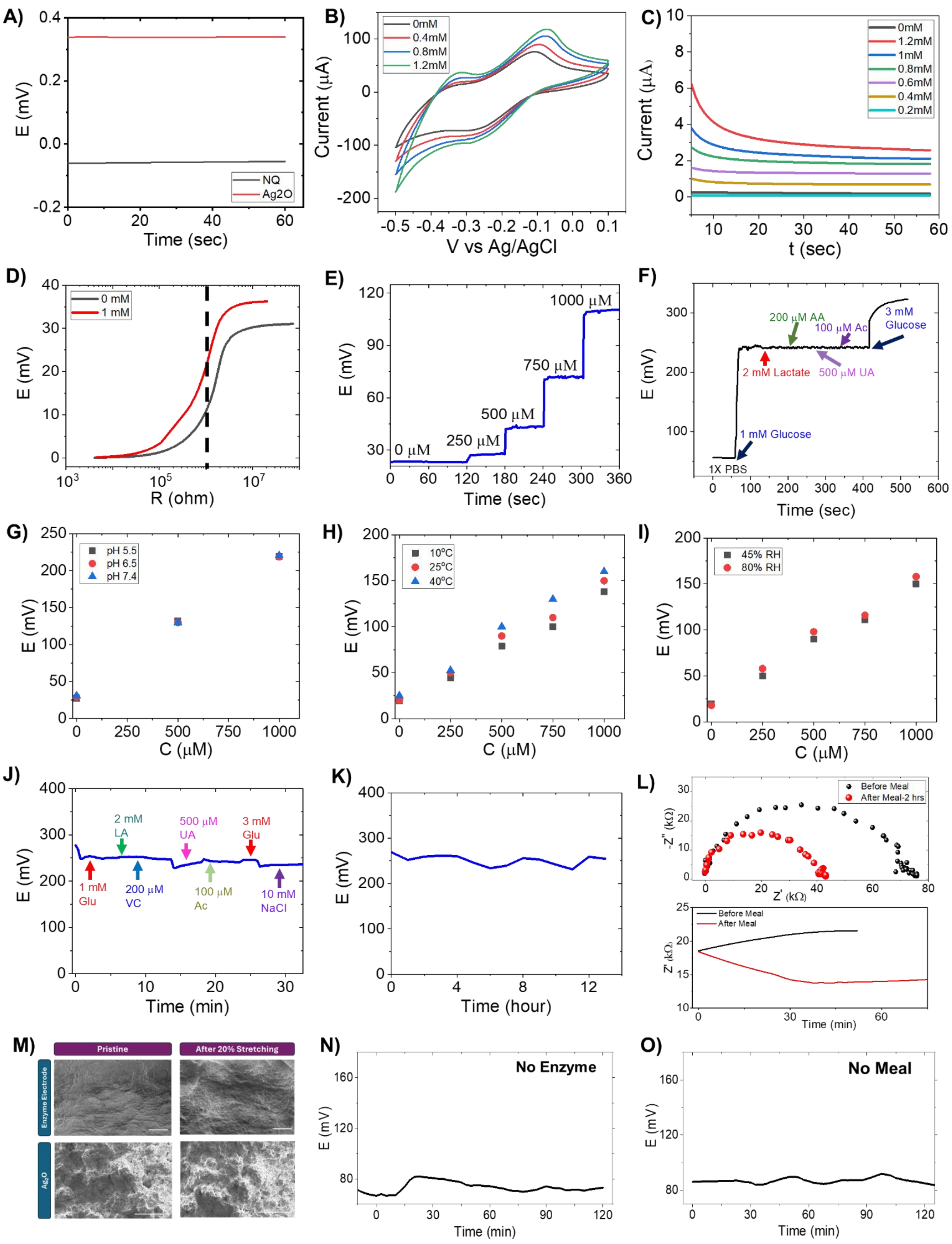
A)
E (mV)
Time (sec)
NQ
Ag2O
B)
Current (μA)
V vs Ag/AgCl
0mM
0.4mM
0.8mM
1.2mM
C)
Current (μA)
t (sec)
0mM
1.2mM
1mM
0.8mM
0.6mM
0.4mM
0.2mM
D)
E (mV)
R (ohm)
0 mM
1 mM
E)
E (mV)
Time (sec)
0 μM
250 μM
500 μM
750 μM
1000 μM
F)
E (mV)
Time (sec)
1X PBS
1 mM Glucose
2 mM Lactate
200 μM AA
100 μM Ac
500 μM UA
3 mM Glucose
G)
E (mV)
C (μM)
pH 5.5
pH 6.5
pH 7.4
H)
E (mV)
C (μM)
10°C
25°C
40°C
I)
E (mV)
C (μM)
45% RH
80% RH
J)
E (mV)
Time (min)
1 mM Glu
2 mM LA
200 μM VC
500 μM UA
100 μM Ac
3 mM Glu
10 mM NaCl
K)
E (mV)
Time (hour)
L)
-Z" (kΩ)
Z' (kΩ)
Before Meal
After Meal-2 hrs
Z' (kΩ)
Time (min)
Before Meal
After Meal
M)
Pristine
After 20% Stretching
Enzyme Electrode
Ag2O
N)
No Enzyme
E (mV)
Time (min)
O)
No Meal
E (mV)
Time (min)

Fig. S1: Electrochemical characterization of glucose sensor and on-body control tests: A) OCP stability of the anode and cathode vs Ag/AgCl reference. B) Cyclic voltammetry of NQ-GOx electrode at 50 mV/sec with glucose additions covering the physiological range in sweat. C) Chronoamperometry validation with glucose additions at -0.1V. D) Linear sweep voltammetry (LSV) trend at 1mV/sec with 0 and 1mM glucose additions, proving that 1MΩ external resistance can distinguish between the lowest and highest glucose concentration in sweat with high resolution. E) Potentiometric calibration plot under 1MΩ external resistance. (F) Interference study of full cell vs. other common sweat analytes. AA: Ascorbic acid, UA: Uric acid, Ac: Acetaminophen G) pH stability validation under varying glucose concentrations. H) Response under varying physiological temperature I) Response under varying humidity. J) Interference test of the Ag_2O electrode vs. Ag/AgCl reference against common sweat analytes. K) Long term stability of the Ag_2O electrode vs. Ag/AgCl reference in 0.1M PBS. L) Bode plot from fingertip before and after two hours past meal intake and Z' vs. t plot from fingertip at 50 kHz showing before and after meal trends. M) SEM images showing the stability of enzymatic and Ag_2O electrode with 20% stretching. Scale: 50 µm (top row), 100 µm (bottom row). N) Control test of the sensor with no enzyme and (O) without meal on the fingertip.

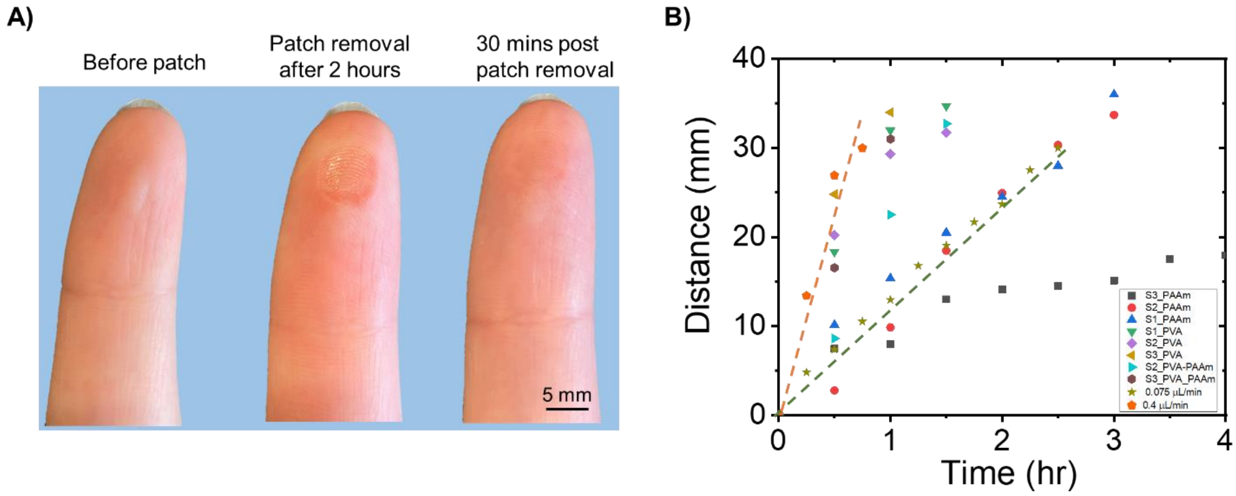

Fig. S2: Hydrogel effect on fingertip and estimation of fingertip sweat rate with validation using a syringe pump. (A) Images confirming no adverse effect on skin after interfacing it with EG treated hydrogel. The skin recovers to its initial condition within ~ 30 minutes after the patch removal. (B) The scattered points denote subject data, and the dotted lines denote syringe pump data. The range is from 75-400 nL/min, in which the PVA-PAAm gel showed sweat rate ~ 200-

300 nL/min. All profiles follow the Lucas Washburn profile ($L(t) = k\sqrt{t}$). The velocity is calculated from the linear regime. S1: Subject 1, S2: Subject 2, S3: Subject 3.

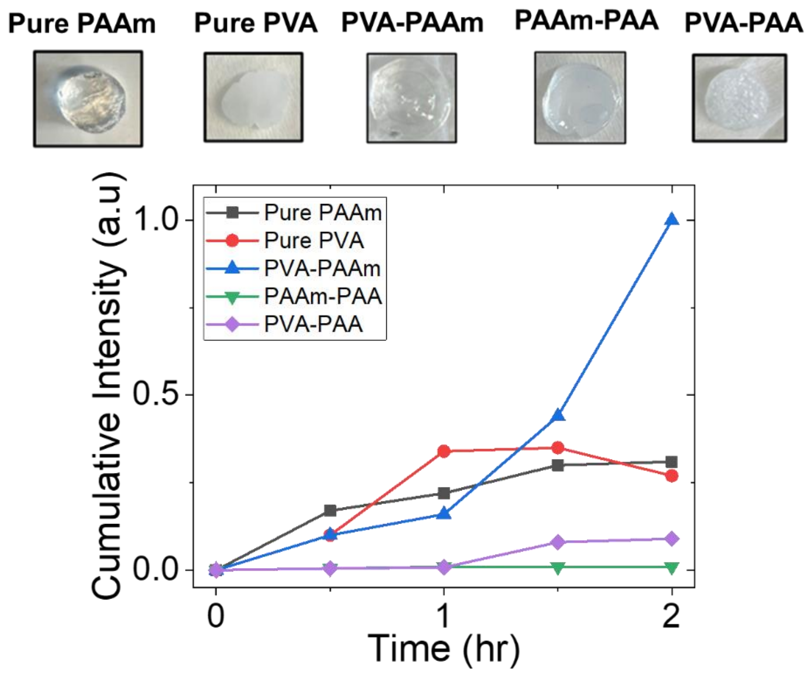

Fig. S3: Cumulative dye intensity trend on paper from the fingertip test with all osmotic hydrogel variants for two hours. The y-axis is normalized with respect to the maximum intensity value. Results show that PVA-PAAm gel works the best on the fingertip, followed by pure PAAm gel.

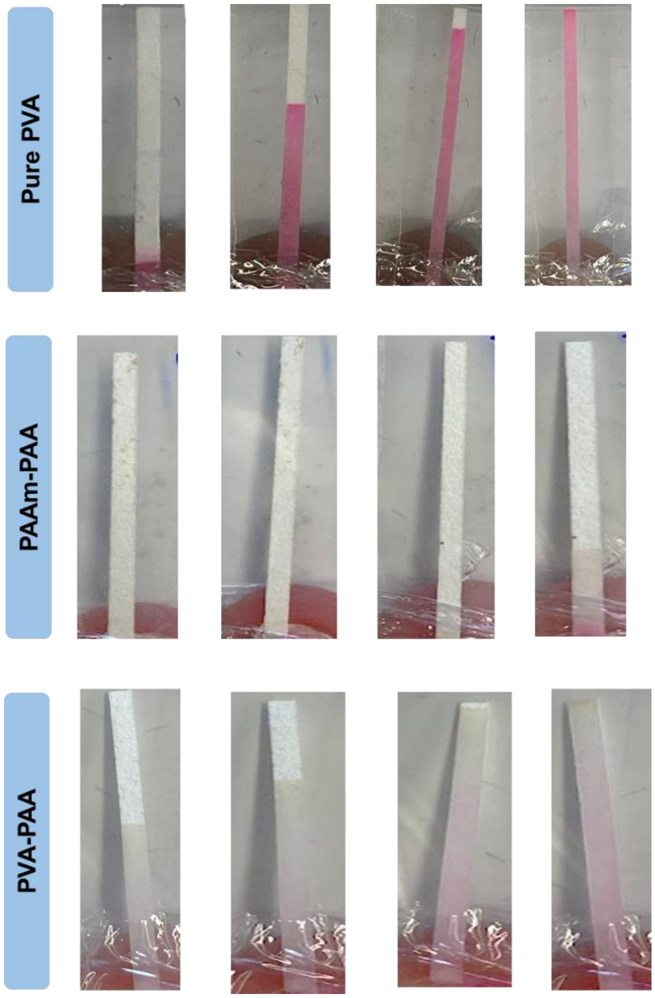

Fig. S4: Dye penetration of EG hydrogels (osmotic + natural) from fingertip. Channel width: 2 mm. Pure PVA hydrogel shows diluted dye flow vs. PVA-PAAm and hence was not used for on-body trials.

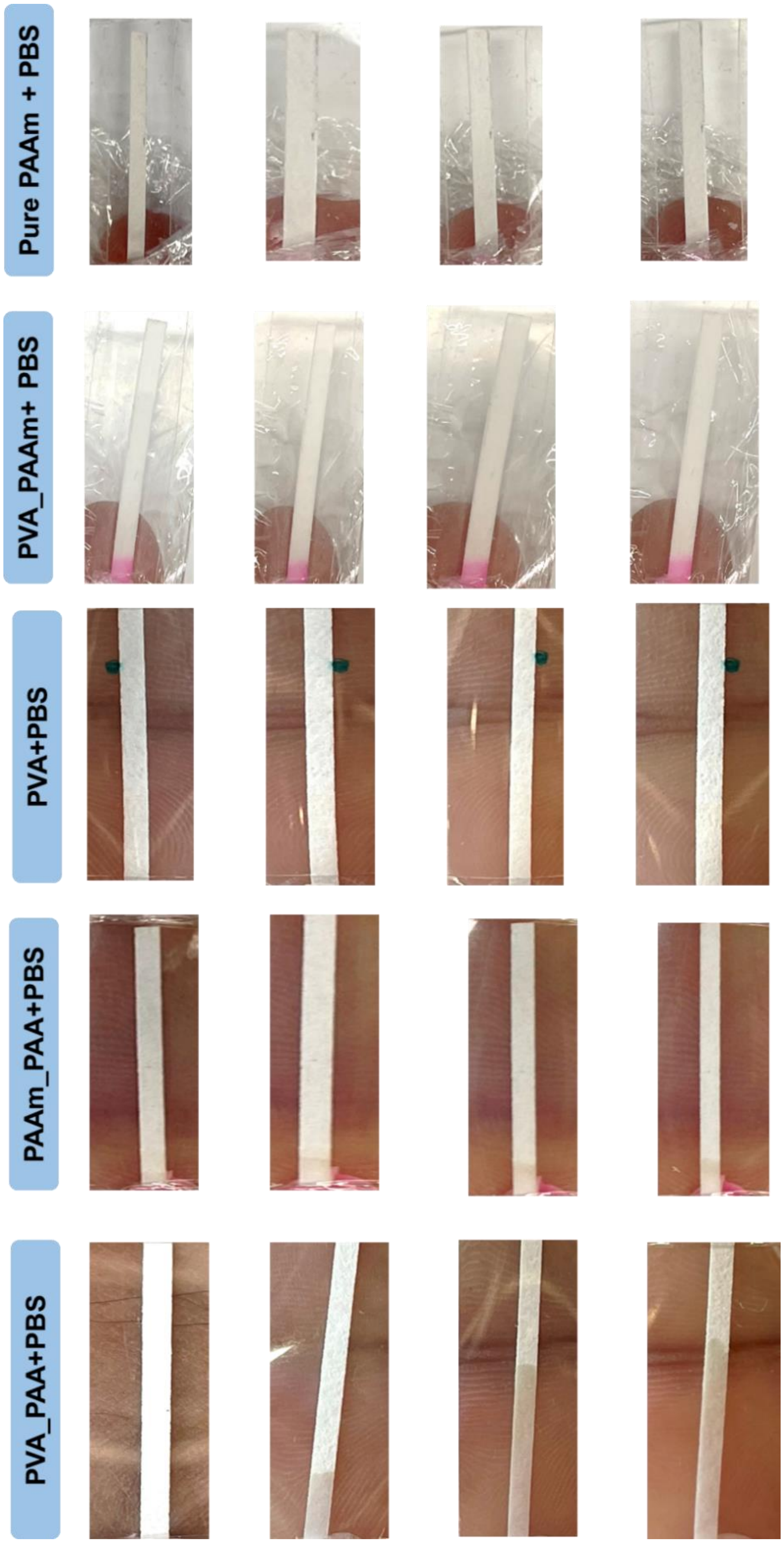

Fig. S5: Dye penetration of PBS hydrogels (only natural perspiration) from fingertip. PBS hydrogels show lesser dye collection vs. EG treated hydrogels. Channel width: 2 mm.

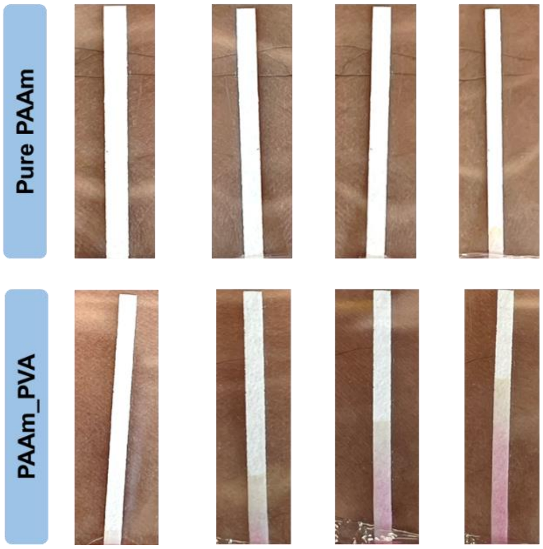

Fig. S6: Dye penetration of EG treated pure PAAm and PVA-PAAm hydrogels from the forearm. Channel width: 2 mm. Although PAAm_PAA hydrogel leads to greater dye in flow on paper, the data from Fig. 2f proves PAAm hydrogel to be better for operating at the forearm.

Supplementary Note 2: Flux analysis with osmotic sampling

Case 1: The sweat rate reported under passive perspiration from the fingertip during continuous on-body trials is reported ~ $3-10\ nL.min^{-1}.cm^{-2}$[1], while with the inclusion of osmotic effect and paper channel, the flow rate ranges ~ $60-85\ nL.min^{-1}.cm^{-2}$. This proves that the osmotic effect can enhance flux intake by at least 5-6 times.

Case 2: Reported passive sweat rate: $0.5\frac{nl}{min}/gland$.[3]

a. **Fingertip:** Sweat gland density: $\sim 350\ \frac{glands}{cm^2}$ [4]; Calculated flow rate from Fig. 2d: ~ $300\ \frac{nL}{min}$; Total extraction area: $0.5\ cm^2$. Therefore, calculated sweat extraction rate ~ $1.7\ \frac{nl}{min}/gland$

b. **Forearm:** Sweat gland density: $\sim 110\ \frac{glands}{cm^2}$; Calculated flow rate from Fig. 2d: ~ $100\ \frac{nL}{min}$; Therefore, calculated sweat extraction rate ~ $1.8\ \frac{nl}{min}/gland$

Thus, osmotic effect in our case can increase the sweat extraction rate per unit gland by ~ 3-4 times vs. natural perspiration, without causing any excessive dilution of sweat glucose.[5,6]

Supplementary Note 3: Theoretical estimation of hydrogel osmotic pressure

The osmotic pressure of a hydrogel is derived theoretically by estimating the swelling pressure using the Flory-Rehner Theory. The swelling capacity of any gel system upon fluid intake is highly dependent on the overall contributions from elastic forces, polymer-solvent interactions, and ionic composition. These effects depend on several thermodynamic properties (*e.g.,* temperature, pH, solution ionic strength and composition). Hence, it is important to estimate each contribution to understand the overall swelling behavior and fluid extraction capacity of a hydrogel.[7–10]

The total swelling pressure (π_{tot}) is derived using the following equation:

$$\pi_{tot} = -V_1^{-1}\left(\frac{\partial \Delta F_{tot}}{\partial n_1}\right) = \pi_{el} + \pi_{mix} + \pi_{ion} \quad (2)$$

where V_1 is the solvent molar volume (18 ml/mol), n_1 is the number of moles of solvent, π_{tot} is the total gel swelling pressure and π_{el}, π_{mix} and π_{ion} are the elastic, mixing and ionic contributions to the total swelling pressure, respectively.

We first quantified the elastic contributions (π_{el}) for all our hydrogel systems. For every hydrogel variant, we estimated its weight swelling ratio (q_p), polymer volume fraction after equilibration ($\emptyset_2$), and gel shear modulus (G) via experiments. These parameters were used for the estimation of π_{tot}. The q_p was calculated as:

$$q_p = \frac{m_{gel}}{m_{dry}} \quad (3)$$

where m_{gel} is the initial mass of gel after equilibration in the osmolyte and m_{dry} is the mass of gel after drying. $\emptyset_2$ was calculated from q_p as follows:

$$\emptyset_2 = \left[1 + \frac{(q_p - 1)\rho}{d}\right]^{-1} \quad (4)$$

where ρ is the polymer density (1.35 g/ml-PAAm, and 1.29 g/ml- PVA)[11] and d is the density of water (1 g/ml). $\emptyset_2\ (PAAm) = 0.68$ and $\emptyset_2\ (PVA - PAAm) = 0.18$.

The elastic contribution in the hydrogel system using $\emptyset_2$ was calculated as follows:

$$\pi_{el} = -G, \tag{5}$$

$$G = G_{dry}\emptyset_2^{1/3} = \frac{E_{dry}}{3}\emptyset_2^{1/3} \tag{6}$$

where G_{dry} and E_{dry} are the dry gel shear modulus and dry elastic modulus, respectively, unequilibrated in any solution.[12] Experimentally (using Mark 10 Material Tester) obtained $\emptyset_2$ and G_{dry}were plugged in eq. (6) to obtain G.

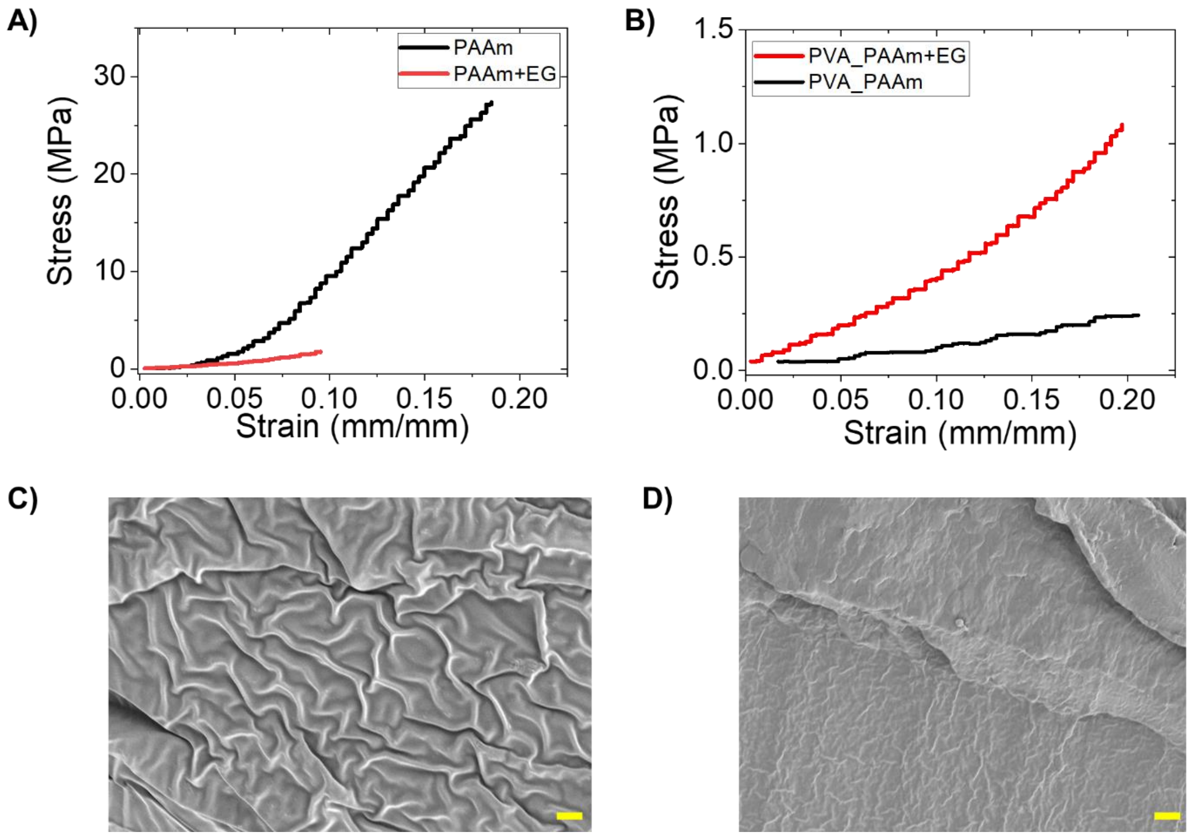

Fig. S7: Mechanical testing and SEM images of hydrogels. Compressive stress vs. strain analysis of (A) pure PAAm and (B) PAAm-PVA hydrogel with and without EG treatment. SEM images of (C) pure PAAm and (D) PAAm-PVA hydrogel after EG treatment. Scale bar: 2 µm.

E_{dry} was measured from the slope (till strain rate 0.05 mm/mm) in Fig. S7 for both hydrogels, which were estimated to be 32 MPa and 3.6 MPa, respectively.

The contribution from the polymer-solvent mixing interactions was calculated as follows:

$$\pi_{mix} = \frac{-RT}{V_1}[\ln(1 - \emptyset_2) + \emptyset_2 + \chi\emptyset_2^2] \tag{7}$$

where χ is the Flory-Huggins polymer-solvent interaction parameter. For hydrogels, $\chi = 0.48$.[13]

The contributions from the ionic interactions were neglected as the hydrogels were not treated with any ionic solvent during the test.

Based on this, $\pi_{tot} = 23.5\ MPa$ (Pure PAAm) and $\pi_{tot} = 0.2\ MPa$ (PVA-PAAm) were estimated.

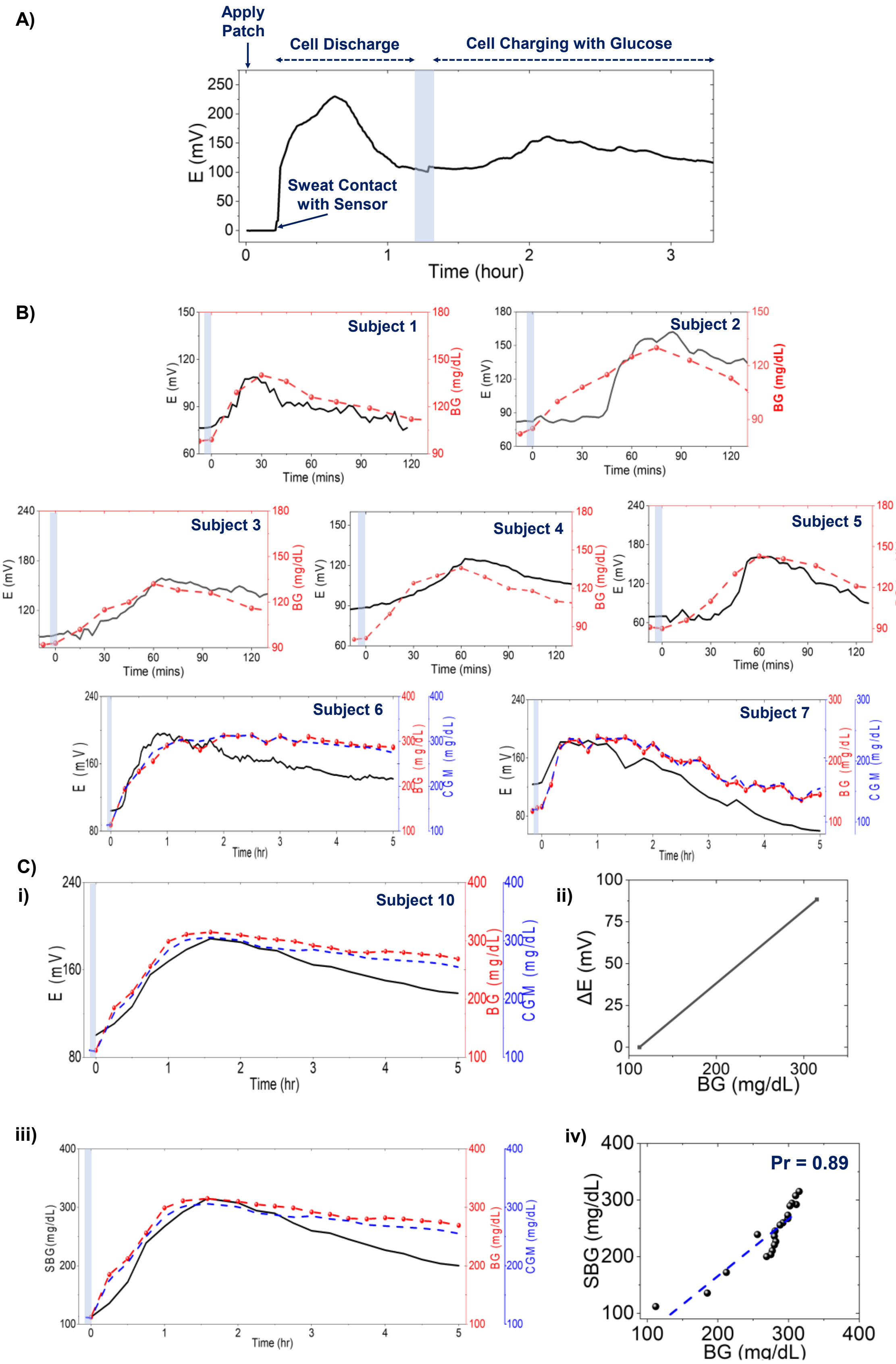
A)
Apply Patch
Cell Discharge
Cell Charging with Glucose
Sweat Contact with Sensor
E (mV)
Time (hour)
B)
Subject 1
Subject 2
Subject 3
Subject 4
Subject 5
Subject 6
Subject 7
E (mV)
BG (mg/dL)
CGM (mg/dL)
Time (mins)
Time (hr)
C)
i)
Subject 10
E (mV)
BG (mg/dL)
CGM (mg/dL)
Time (hr)
ii)
ΔE (mV)
BG (mg/dL)
iii)
SBG (mg/dL)
BG (mg/dL)
CGM (mg/dL)
Time (hr)
iv)
Pr = 0.89
SBG (mg/dL)
BG (mg/dL)

Fig. S8: Sensor data from fingertip. (A) A typical discharge-charge profile of the glucose sensor from fingertip. (B) $E(t)$ vs. t plots from fingertip of 5 healthy subjects (red and black) and 2 diabetic subjects (black and blue). (C) Additional data from a diabetic subject (Subject #10) showing the blood, CGM and (i) potential data (ii) ΔE vs. BG calibration ($m = 0.42\ \frac{mV}{mg/dL}$ and $c = -48.7\ mV$), (iii) $SBG(t)$ profile, and (iv) correlation plot of $SBG(t)$ vs. $BG(t)$. Pr=0.89 and MARD = 12.7%. Blue: Meal intake.

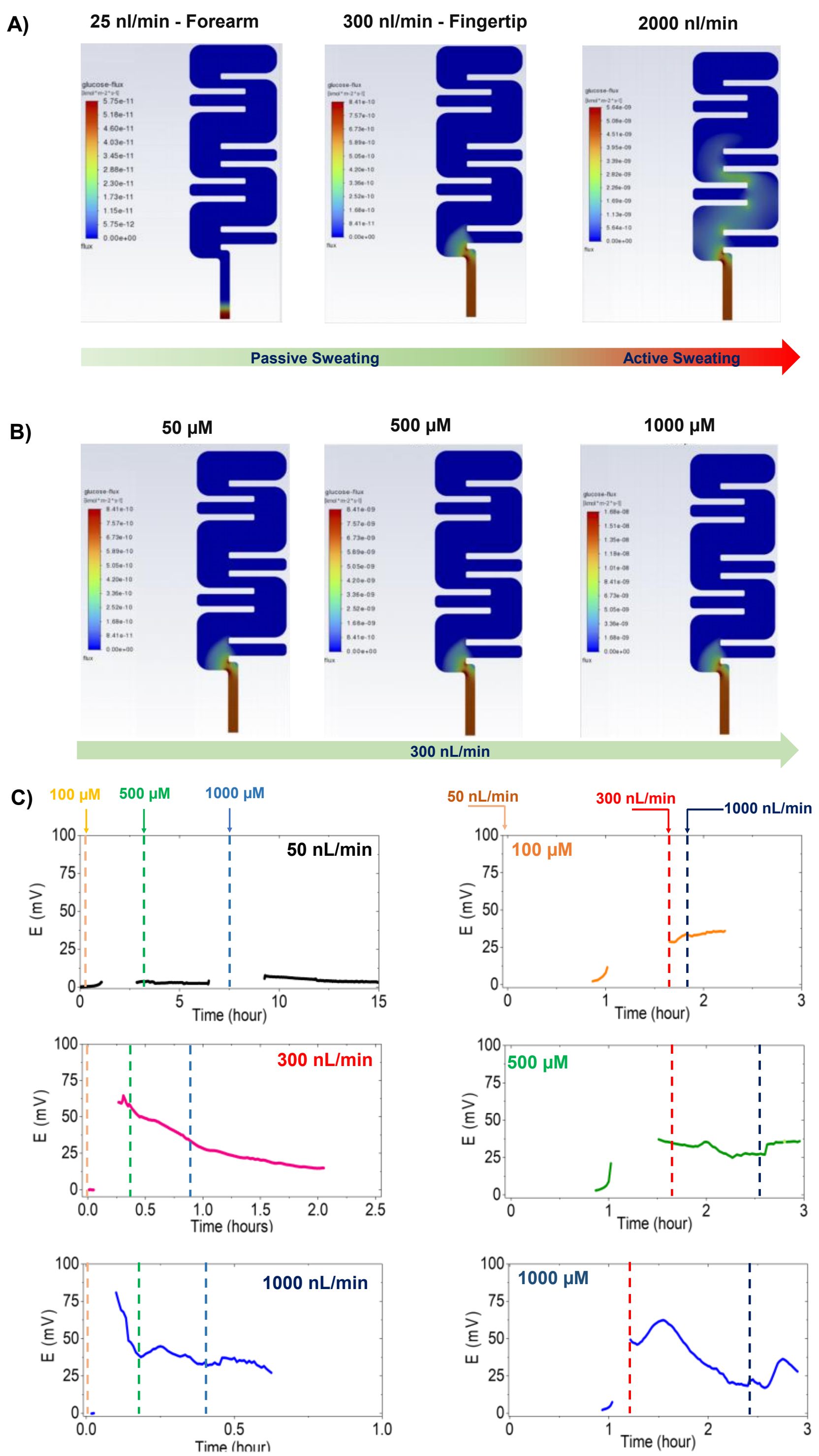

Fig. S9: Finite element simulations and in-vitro study of flow rate effects. (A) Finite element analysis of glucose flux profile in the paper microfluidic channel under varying sweat rates with an inlet glucose concentration of 50 µM (assumed glucose concentration at rest before meal). (B) Flux profile at fingertip flow rate under varying inlet glucose concentrations. Simulation time: 10

minutes. (C) First column: Sensor response under varying inlet glucose concentrations at constant flow rates. At low flow rates (<100 nl/min), glucose concentration is mass transfer limited, while at flow rates greater than 100 nl/min, the sensor will not be mass transfer limited. High flow rates (> 1µL/min) make the system more sensitive to convective effects. Second column: Sensor response under varying inlet flow rates at constant glucose concentration. The convective effects (sweat rate >1 µl/min) affect the response more when high glucose (>500 µM) already exists in the channel. Results also verify negligible impact of the sensor output at the osmotic fingertip and forearm sweat rate. Sweat rate corrections would be needed only under high flow rates.

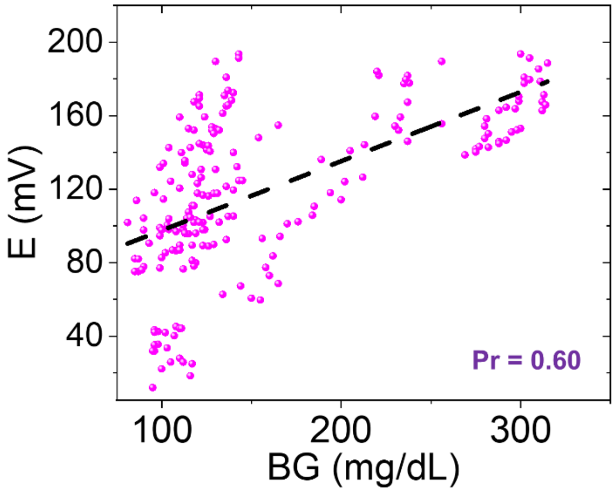

Fig. S10: Correlation of potential data from fingertip vs. BG. $E(t)$ vs. BG of all subjects (n=208) from fingertip sweat.

Supplementary Note 4: Calibration model to acquire BG dynamics from $E(t)$

The two-point calibration model is developed by plotting minimum and maximum values of $\Delta E(t)$ vs $BG(t)$. The basis of this assumption is:

1. When $\Delta E(t) = 0$, $BG(t) = BG_f$
2. $SBG(t) \rightarrow BG(t)$ will give minimum mean absolute relative difference (MARD).

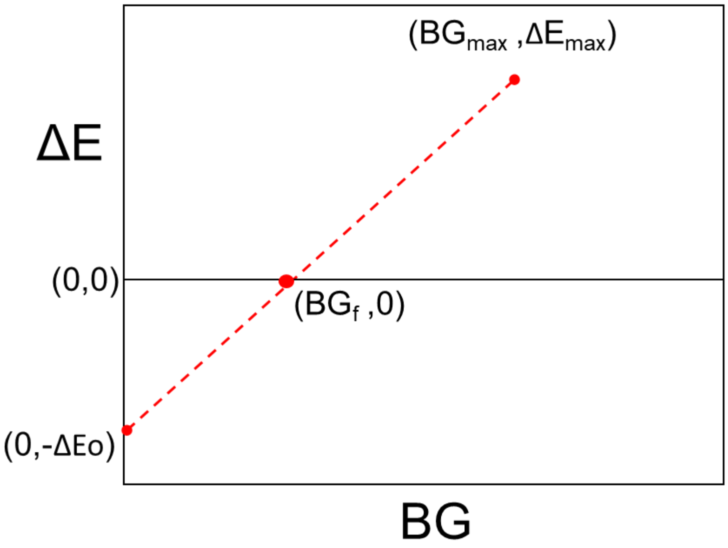

From the calibration curve, the corrected slope (m) is given by:

$$m = \frac{\Delta E_{max}}{BG_{max} - BG_f}$$

and $$SBG\ (t) = \frac{E(t) - E_0 + \Delta E_0}{m}$$

After substituting all values, the final derived equation to convert $E(t)$ is:

$$\boldsymbol{SBG(t) = (\frac{E(t) - E_0 + \Delta E_0}{\Delta E_{max}})(BG_{max} - BG_f)}$$

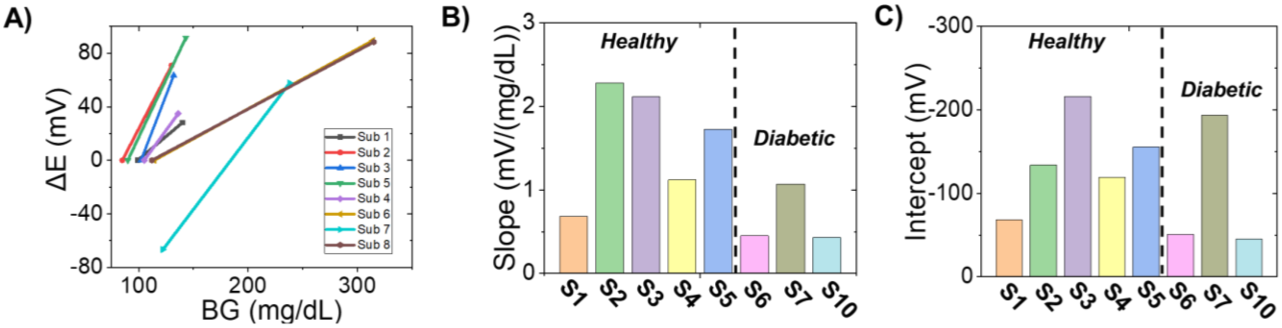

Fig. S11: Slope and intercept analysis from fingertip sweat. Plots showing the (A) two-point calibration of all subjects, (B) corrected slope values, and (C) corrected intercept values for healthy

and diabetic subjects with fingertip sweat. The stability of these parameters with multiple trials and days will determine the long-term stability of $SBG\ (t)$ values.

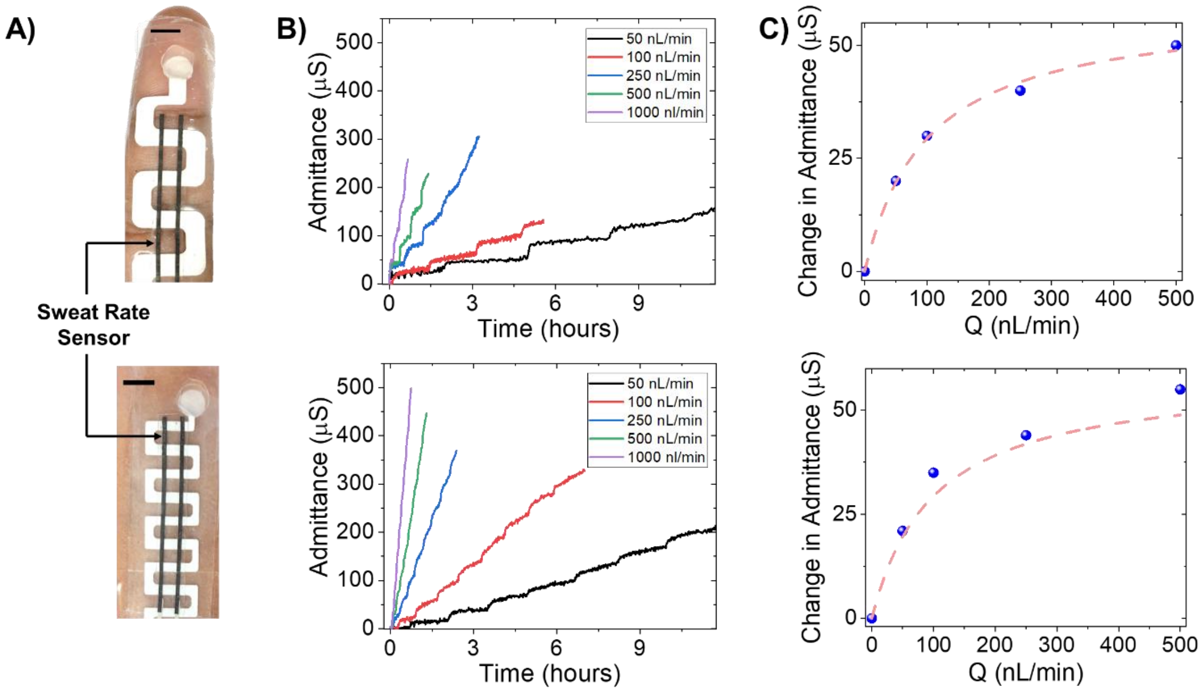

Fig. S12: Sweat rate sensor integration with SCGM. (A) Sweat rate sensor integrated with paper microfluidics to operate with fingertip and forearm sweat. (B) Admittance profile under different flow rates of 0.1M PBS, and (C) Calibration plot of the admittance change vs. flow rate. The paper channel for fingertip sweat rate measurement has a greater width to accommodate and measure from higher sampled sweat volume. The forearm channel is narrow so that lesser sweat flowing in the channel can instantaneously encounter the sweat rate sensor and show an admittance change. Top row: fingertip, Bottom row: forearm. Scale bar: 5 mm.

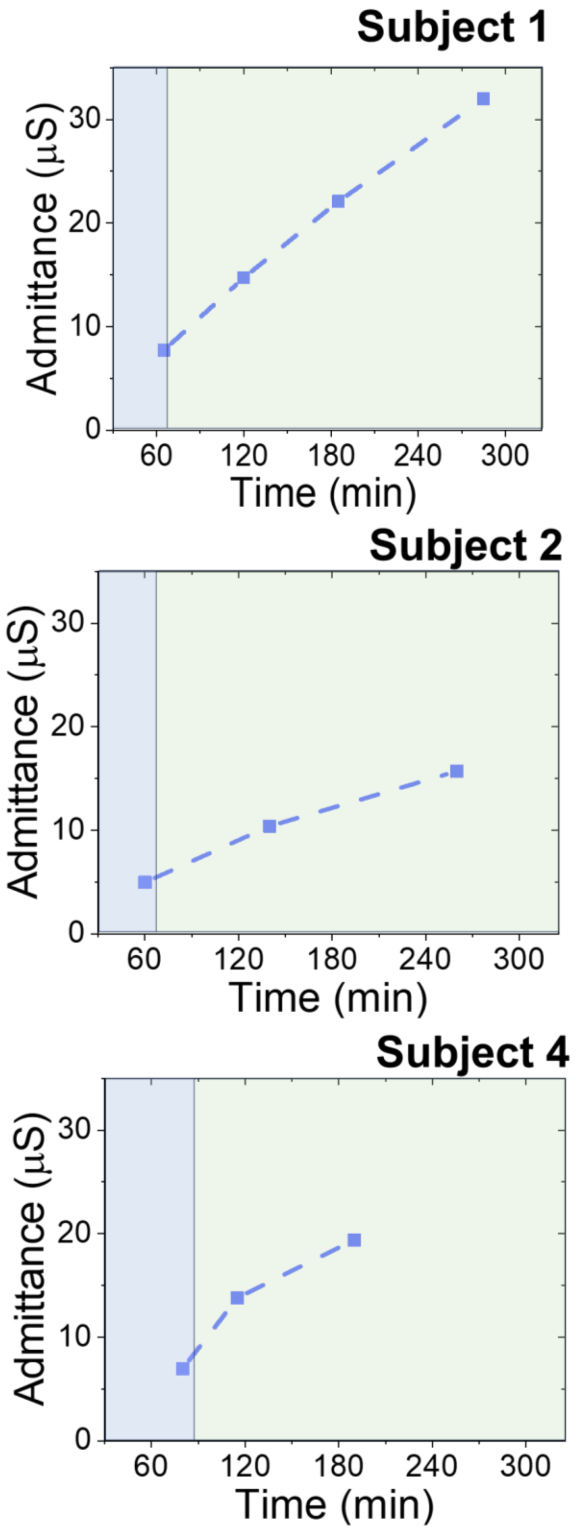

Fig. S13: Sweat rate analysis from fingertip. Admittance vs time profile for three healthy subjects from fingertip sweat. Blue region: before meal, Green: after meal. The sweat rate can be estimated using the calibration plot in Fig. S12 top row.

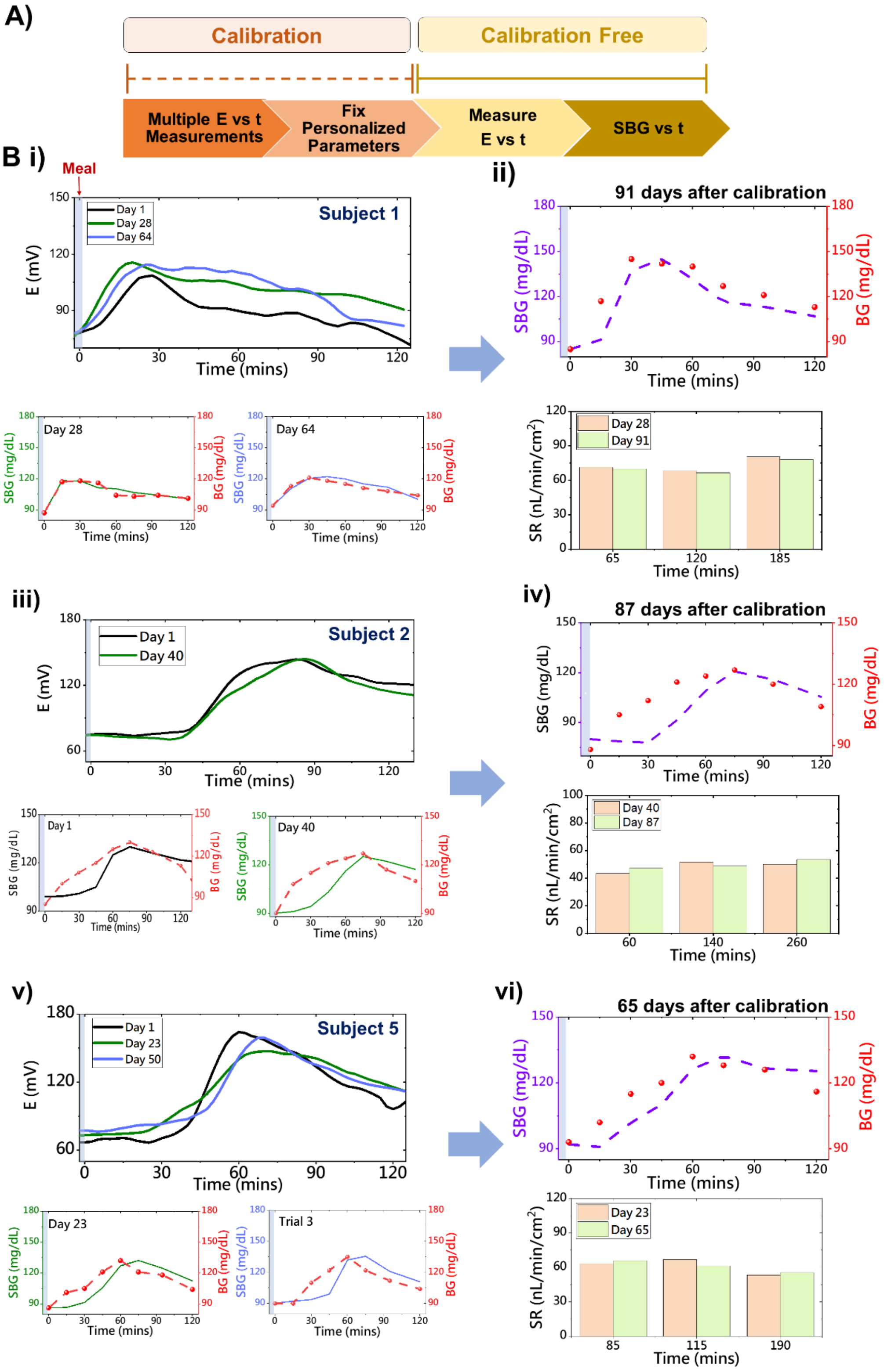

Fig. S14: Stability analysis of personalized calibration models with fingertip sweat. (A) Schematic highlighting the steps towards achieving a calibration-free approach for $SBG(t)$

estimation. With multiple trials on different days, the variations in the personalized parameters were first studied. If found similar, these values are then fixed for each subject and used to directly convert $E(t)$ to $SBG(t)$ without measuring $BG(t)$ frequently. B i,ii) Plot showing the stability of $E(t)$ vs. t over multiple trials conducted over different days for subject 1 with fingertip sweat. $BG(t)$ is also evaluated to check MARD. With three trials (till day 64), the personalized parameters (m, c) remained stable and were fixed and used on the 91st day after the initial calibration to get the $SBG(t)$ profile. $BG(t)$ on the 91st day was also measured as a reference to check MARD, but not used to derive the personalized parameters. A similar approach was executed for Subject 2 (B iii,iv) to get $SBG(t)$ after 87 days from initial calibration and on Subject 5 (B v,vi) to get $SBG(t)$ after 65 days post calibration. All sweat rate values are estimated using the design in Fig. S12 top row, which showed no effect on the $SBG(t)$ profile.

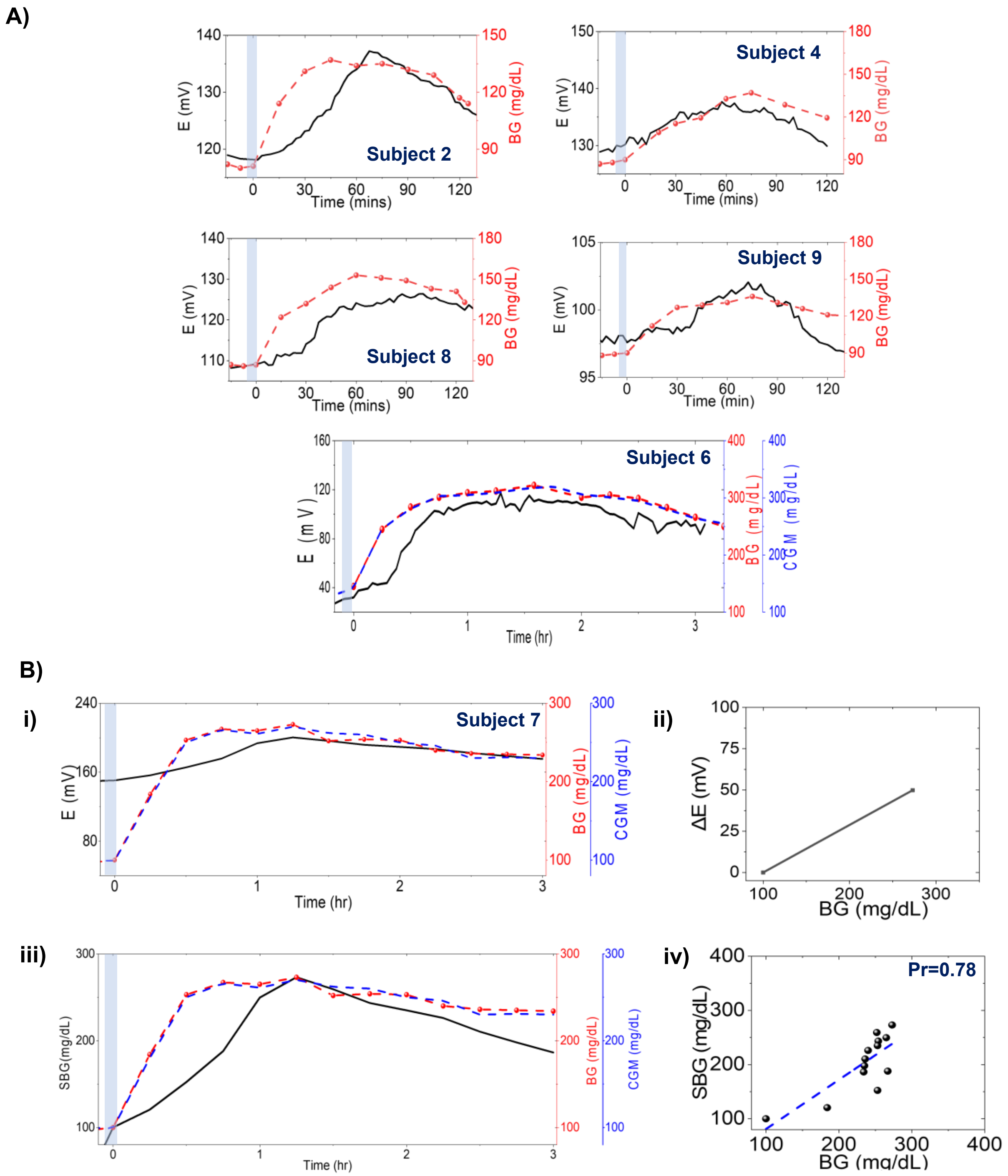

Fig. S15: Sensor data from forearm. A) $E(t)$ vs. t plots from forearm of 4 healthy subjects (red and black) and 1 diabetic subject (black and blue). B) Additional data from a diabetic subject (Subject #7) showing the blood, CGM and (i) potential data (ii) ΔE vs. BG calibration ($m =$

$0.30\ \frac{mV}{mg/dL}$ and $c = -28.8\ mV$), (iii) $SBG(t)$ profile, and (iv) correlation plot of $SBG(t)$ vs. $BG(t)$. Pr=0.78 and MARD = 13.6%. Blue: Meal intake.

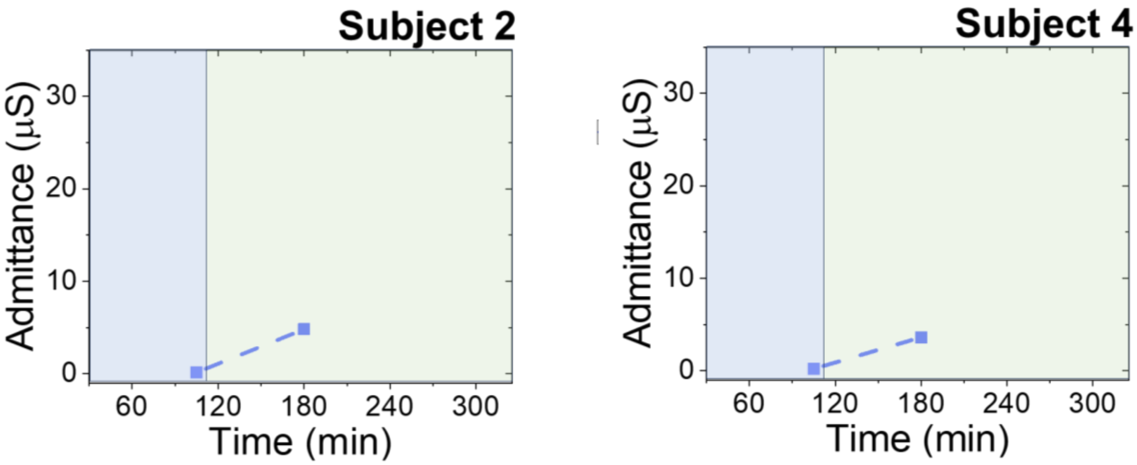

Fig. S16: Sweat rate analysis from forearm. Admittance vs time profile of two healthy subjects from forearm sweat. Blue region: before meal, Green: After meal. The sweat rate can be estimated using the calibration plot in Fig. S12 top row.

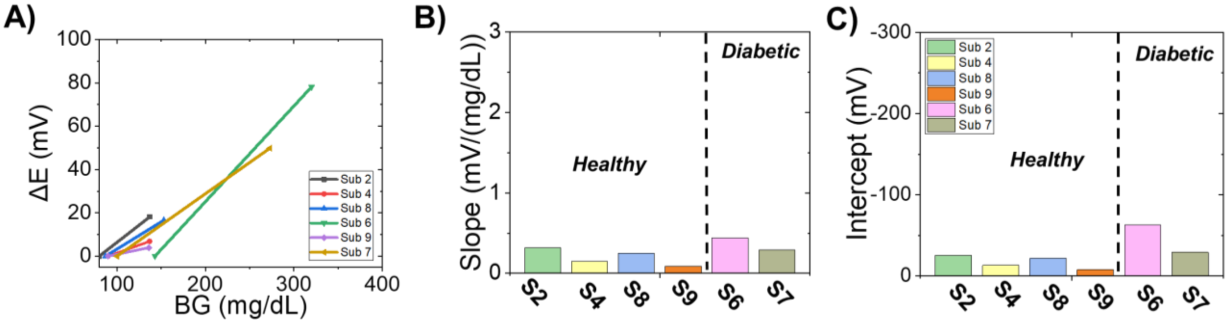

Fig. S17: Slope and intercept analysis from forearm sweat. Plots showing the (A) two-point calibration, (B) corrected slope values, and (C) corrected intercept values for healthy and diabetic subjects with forearm sweat.

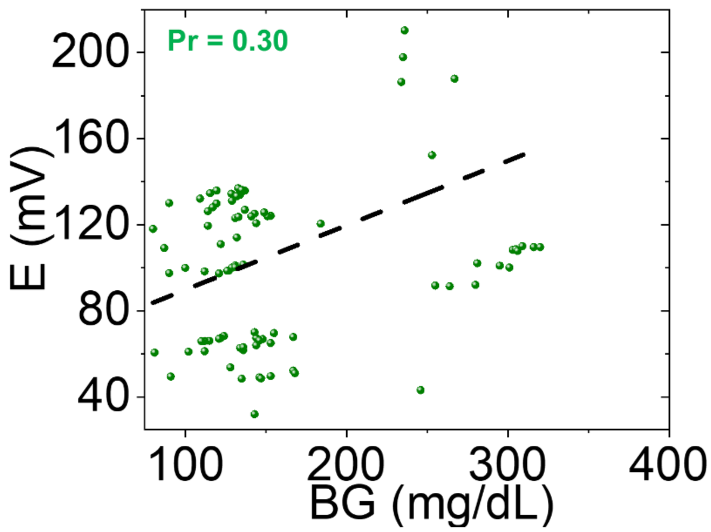

Fig. S18: Correlation of potential data from forearm vs. BG. $E(t)$ vs. BG of all subjects (n=92) from forearm sweat. Pr=0.30 means poor between raw sweat glucose potential and BG.

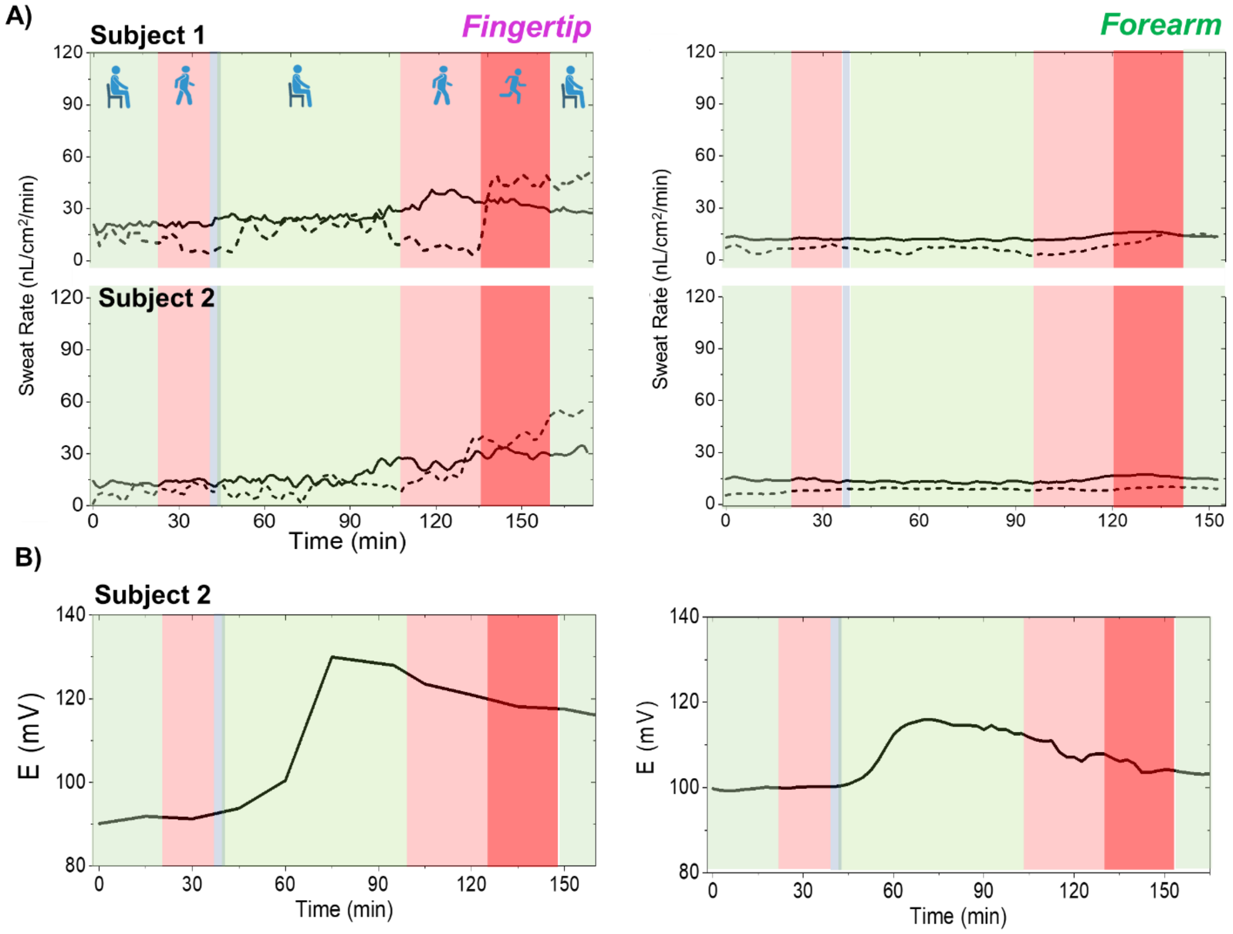

Fig. S19: Sweat rate and E (t) analysis during outdoor activities. A) Sweat rate and vs time from fingertip and forearm during routine activities of sitting and walking. Dotted: 0.1M PBS hydrogel (natural perspiration), solid: with EG hydrogel (natural and osmotic). The osmotic effect reduces the sweat rate variations vs. natural perspiration during outdoor activities, eventually eliminating the necessity of introducing sweat rate-based corrections of $SBG\ (t)$ values. The minor fluctuations reflect motion artifacts which can recover over time. Note: The natural perspiration trend is obtained by removing the signal from PBS only so that only sweat can be tracked. B) $E(t)$ vs t profile with EG hydrogel for subject 2 under varying physiological activities. Green: Rest, Light red: Medium intensity walking, Blue: Meal, Dark red: High intensity walking.

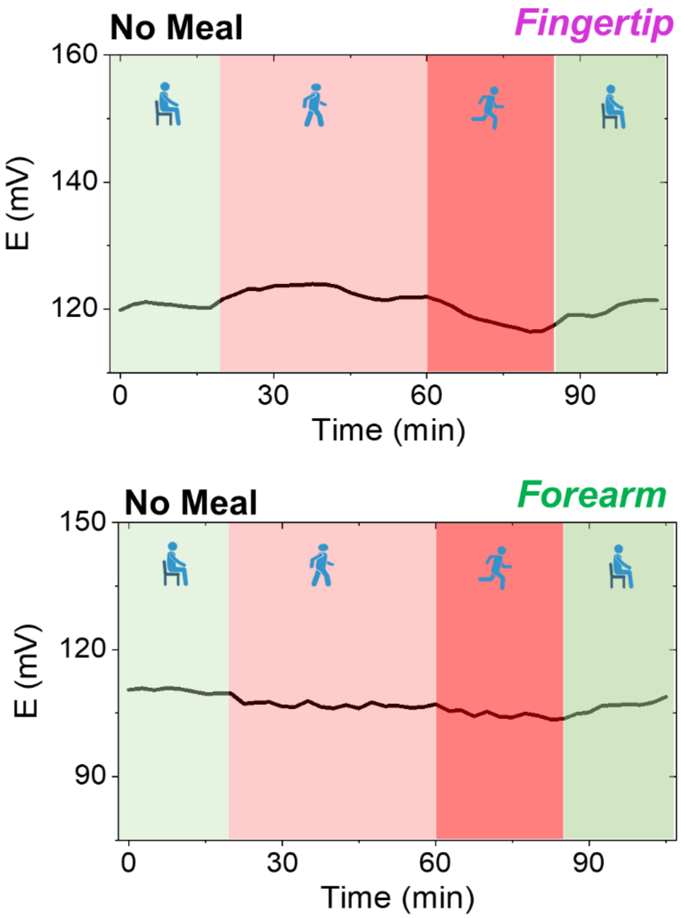

Fig. S20: On-body control studies during outdoor activities. Control test of $E(t)$ vs. t with no meal for fingertip and forearm. No change in response confirms the signal to be coming from glucose.

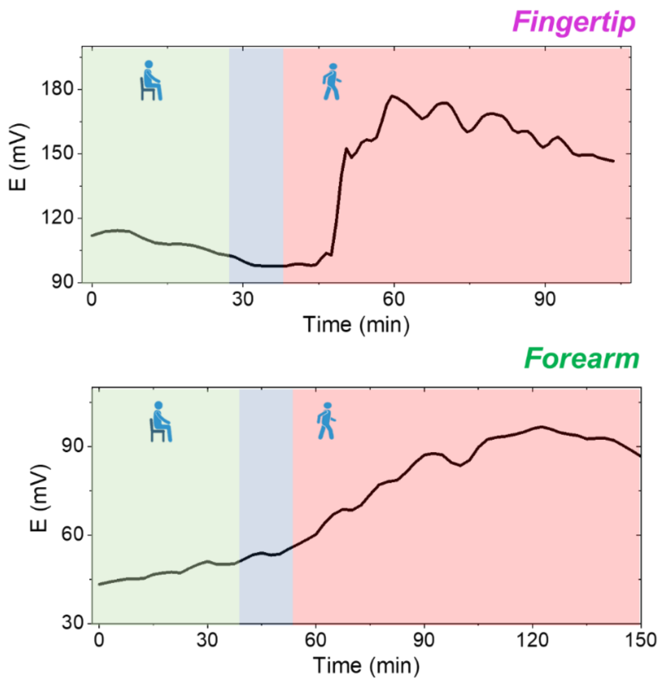

Fig. S21: Data from instantaneous walking after meal consumption during outdoor activities. Potential signals from fingertip and forearm sweat during outdoor testing where the subject started walking right after meal consumption. Green: Rest, Blue: Meal, Red: Medium intensity walking. The continuous increase in potential signal under outdoor walking confirms the response from glucose with minimal interference from sweat rate.

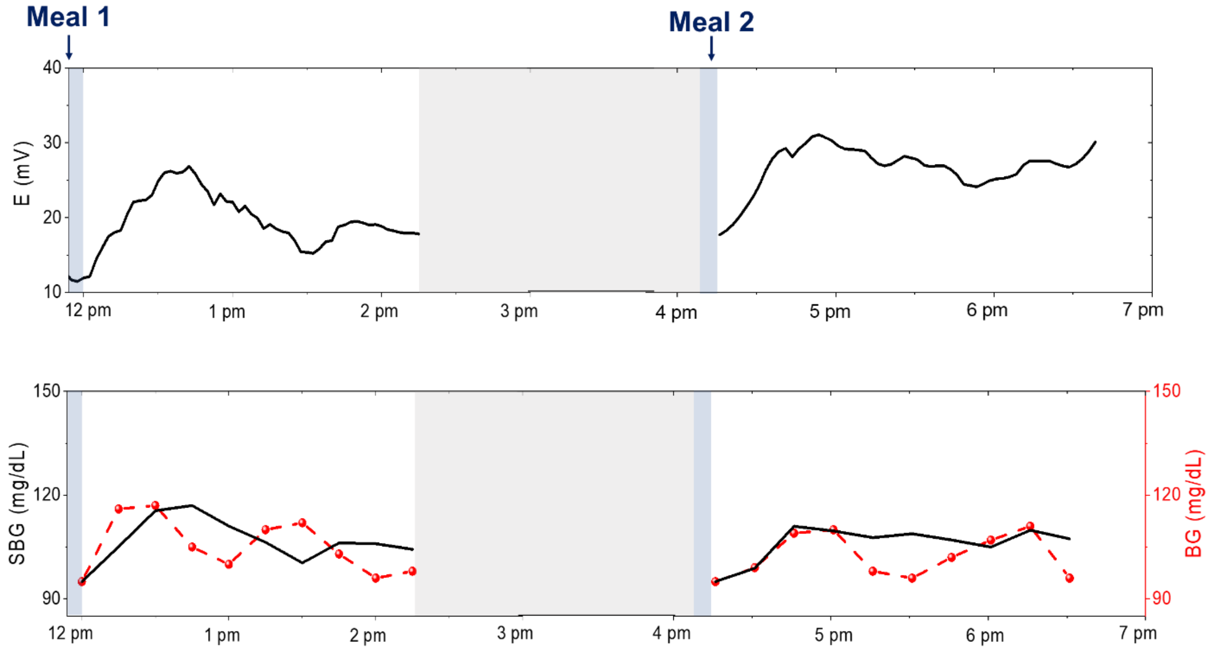

Fig. S22: Fingertip glucose profile (potential and $SBG(t)$) of a subject with multiple meals. Grey zone: Resting duration. The $SBG(t)$ profile followed a diphasic behavior, which is well reported in the field of continuous glucose monitoring.[14,15] The data proves that multiple meals can have varying effects on different individuals. MARD: 6.60%

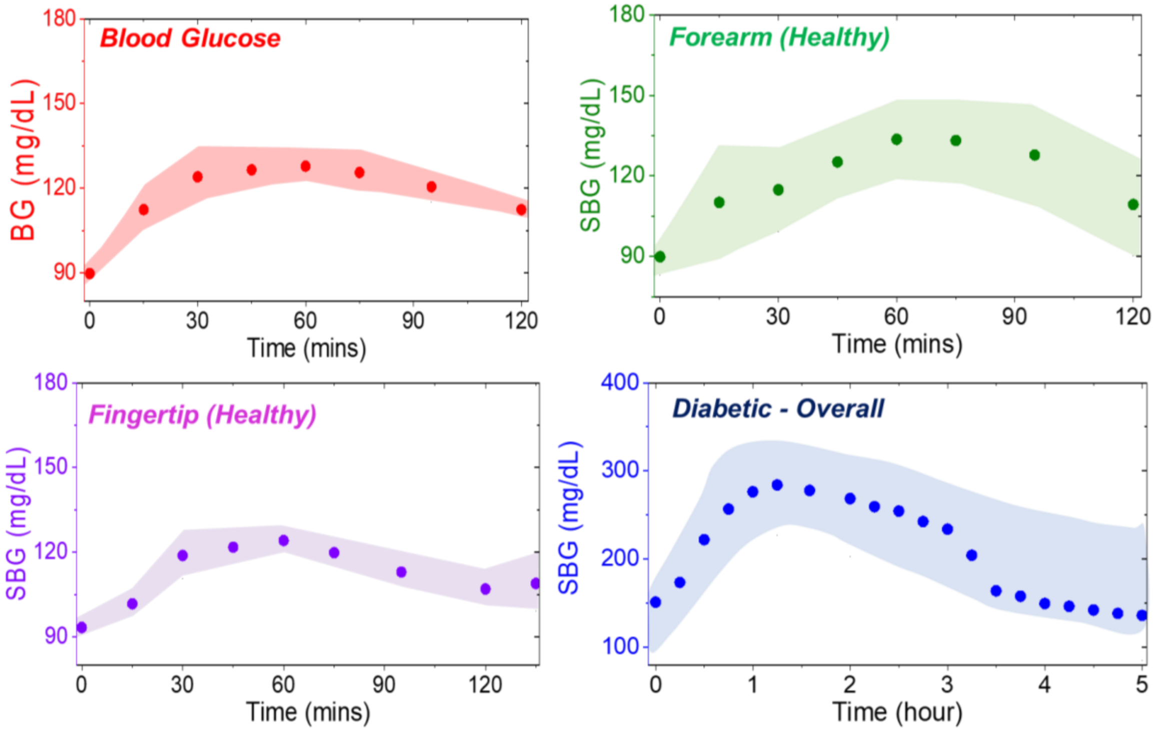

Fig. S23: Overall analysis of $SBG\ (t)$ trends. Plot of mean $SBG\ (t)$ vs. t with shaded 95% confidence interval zone. The narrow interval zone from the fingertip vs. BG profile validates it to be a better location (vs. forearm) for conducting continuous sweat glucose monitoring.

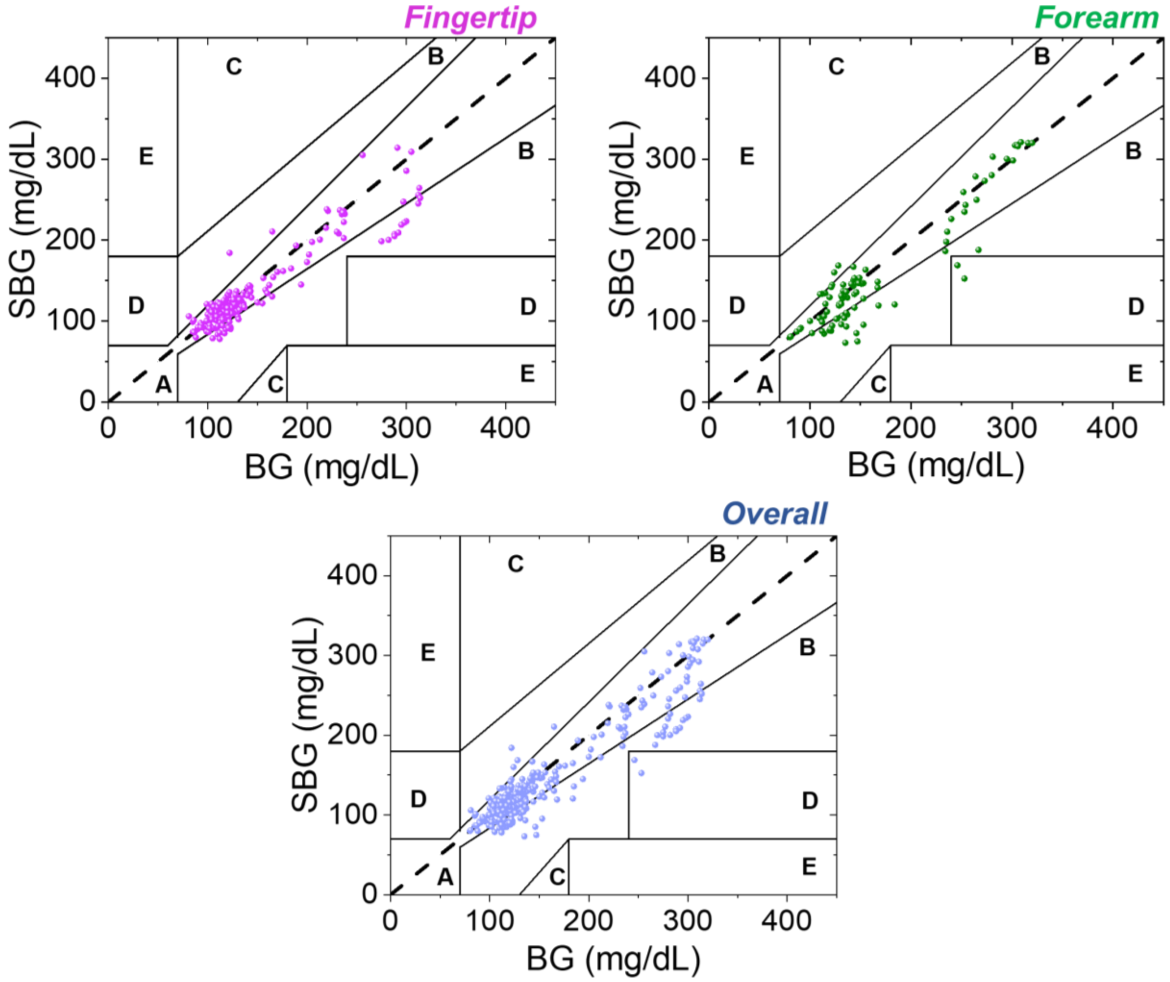

Fig. S24: Clarke's error grid analysis from fingertip and forearm. The overall MARD with n=300 is ~ 11.01 % and 99.3 % of data points lie in the A+B zone. Overall Pr = 0.92.

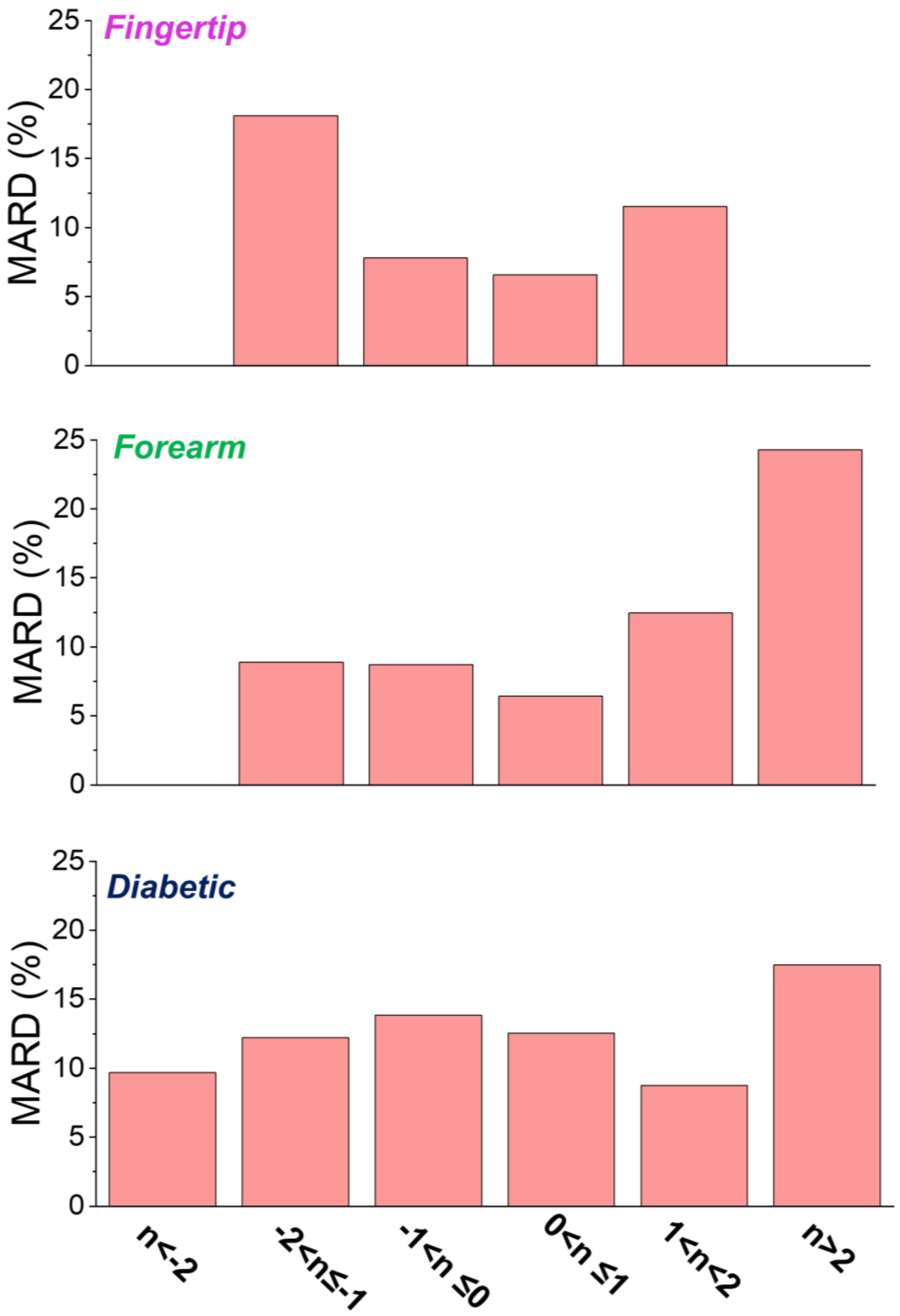

Fig. S25: MARD distribution vs. rate of BG change. MARD analysis across different zones of BG change per unit time (n, mg/dL/min). Fingertip (#subjects =8) and forearm (#subjects= 6) data are from healthy subjects only, while diabetic (#subject=3) plot covers both fingertip and forearm.

References:

[1] H. Y. Y. Nyein, M. Bariya, B. Tran, C. H. Ahn, B. J. Brown, W. Ji, N. Davis, A. Javey, *Nat. Commun.* **2021**, *12*, 1.

[2] Z. Sonner, E. Wilder, J. Heikenfeld, G. Kasting, F. Beyette, D. Swaile, F. Sherman, J. Joyce, J. Hagen, N. Kelley-Loughnane, R. Naik, *Biomicrofluidics* **2015**, *9*, 031301.

[3] T. D. La Count, A. Jajack, J. Heikenfeld, G. B. Kasting, *J. Pharm. Sci.* **2019**, *108*, 364.

[4] N. A. S. Taylor, C. A. Machado-Moreira, *Extrem. Physiol. Med.* **2013**, *2*, 4.

[5] J. Moyer, D. Wilson, I. Finkelshtein, B. Wong, R. Potts, *Diabetes Technol. Ther.* **2012**, *14*, 398.

[6] A. Jajack, M. Brothers, G. Kasting, J. Heikenfeld, *PLoS One* **2018**, *13*, e0200009.

[7] A. Razmjou, Q. Liu, G. P. Simon, H. Wang, *Environ. Sci. Technol.* **2013**, *47*, 13160.

[8] F. Horkay, I. Tasaki, P. J. Basser, *Biomacromolecules* **2000**, *1*, 84.

[9] F. Horkay, P. J. Basser, *Biomacromolecules* **2004**, *5*, 232.

[10] J. P. Baker, L. H. Hong, H. W. Blanch, J. M. Prausnitz, *Macromolecules* **1994**, *27*, 1446.

[11] S. Durmaz, O. Okay, *Polymer (Guildf).* **2000**, *41*, 3693.

[12] D. Caccavo, S. Cascone, G. Lamberti, A. A. Barba, *Chem. Soc. Rev.* **2018**, *47*, 2357.

[13] D. Morales, E. Palleau, M. D. Dickey, O. D. Velev, *Soft Matter* **2014**, *10*, 1337.

[14] O. Tschritter, A. Fritsche, F. Shirkavand, F. Machicao, H. Häring, M. Stumvoll, *Diabetes Care* **2003**, *26*, 1026.

[15] J. Y. Kim, S. F. Michaliszyn, A. Nasr, S. J. Lee, H. Tfayli, T. Hannon, K. S. Hughan, F. Bacha, S. Arslanian, *Diabetes Care* **2016**, *39*, 1431.
